# Multinational Association of Supportive Care in Cancer (MASCC) clinical practice guidance for the prevention of breast cancer-related arm lymphoedema (BCRAL): international Delphi consensus-based recommendations

**DOI:** 10.1016/j.eclinm.2024.102441

**Published:** 2024-02-02

**Authors:** Henry C.Y. Wong, Matthew P. Wallen, Adrian Wai Chan, Narayanee Dick, Pierluigi Bonomo, Monique Bareham, Julie Ryan Wolf, Corina van den Hurk, Margaret Fitch, Edward Chow, Raymond J. Chan, MASCC BCRAL Expert Panel and the Oncodermatology and Survivorship Study Groups, Muna AlKhaifi, Belen Alonso Alvarez, Suvam Banerjee, Kira Bloomquist, Pierluigi Bonomo, Pinar Borman, Yolande Borthwick, Dominic Chan, Sze Man Chan, Yolanda Chan, Ngan Sum Jean Cheng, J. Isabelle Choi, Edward Chow, Yin Ping Choy, Kimberly Corbin, Elizabeth Dylke, Pamela Hammond, Satoshi Hirakawa, Kimiko Hirata, Shing Fung Lee, Marianne Holt, Peter Johnstone, Yuichiro Kikawa, Deborah Kirk, Haruru Kotani, Carol Kwok, Jessica Lai, Mei Ying Lim, Michael Lock, Brittany Lorden, Page Mack, Stefano Magno, Icro Meattini, Gustavo Nader Marta, Margaret McNeely, Tammy Mondry, Luis Enrique Lopez Montoya, Mami Ogita, Misato Osaka, Stephanie Phan, Philip Poortmans, Bolette Skjødt Rafn, Abram Recht, Agata Rembielak, Angela Río-González, Jolien Robijns, Naoko Sanuki, Charles B. Simone, Mateusz Spałek, Kaori Tane, Luiz Felipe Nevola Teixeira, Mitsuo Terada, Mark Trombetta, Kam Hung Wong, Katsuhide Yoshidome

**Affiliations:** a Department of Oncology, Princess Margaret Hospital, Hong Kong S.A.R, China; b Caring Futures Institute, College of Nursing and Health Sciences, Flinders University, Adelaide, Australia; c Department of Clinical Oncology, Tuen Mun Hospital, Hong Kong S.A.R, China; d Department of Radiation Oncology, Azienda Ospedaliero-Universitaria Careggi, University of Florence, Italy; e Flinders Health Medical Research Consumer Advisory Board, Flinders University, South Australia, Australia; f South Australia Lymphoedema Compression Garment Subsidy Advisory Group, South Australia, Australia; g Department of Radiation Oncology, University of Rochester, New York, USA; h R&D Department, Netherlands Comprehensive Cancer Organization (IKNL), Utrecht, Netherlands; i Lawrence S. Bloomberg Faculty of Nursing, University of Toronto, Toronto, Canada; j Department of Radiation Oncology, Sunnybrook Health Sciences Centre, University of Toronto, Toronto, Canada

**Keywords:** Prevention, Breast cancer related arm lymphoedema, Delphi consensus

## Abstract

**Background:**

Developing strategies to prevent breast cancer-related arm lymphoedema (BCRAL) is a critical unmet need because there are no effective interventions to eradicate it once it reaches a chronic state. Certain strategies such as prospective surveillance programs and prophylactic lymphatic reconstruction have been reported to be effective in clinical trials. However, a large variation exists in practice based on clinician preference, organizational standards, and local resources.

**Methods:**

A two-round international Delphi consensus process was performed from February 27, 2023 to May 25, 2023 to compile opinions of 55 experts involved in the care and research of breast cancer and lymphoedema on such interventions.

**Findings:**

Axillary lymph node dissection, use of post-operative radiotherapy, relative within-arm volume increase one month after surgery, greater number of lymph nodes dissected, and high body mass index were recommended as the most important risk factors to guide selection of patients for interventions to prevent BCRAL. The panel recommended that prospective surveillance programs should be implemented to screen for and reduce risks of BCRAL where feasible and resources allow. Prophylactic compression sleeves, axillary reverse mapping and prophylactic lymphatic reconstruction should be offered for patients who are at risk for developing BCRAL as options where expertise is available and resources allow. Recommendations on axillary management in clinical T1–2, node negative breast cancer patients with 1–2 positive sentinel lymph nodes were also provided by the expert panel. Routine axillary lymph node dissection should not be offered in these patients who receive breast conservation therapy. Axillary radiation instead of axillary lymph node dissection should be considered in the same group of patients undergoing mastectomy.

**Interpretation:**

An individualised approach based on patients' preferences, risk factors for BCRAL, availability of treatment options and expertise of the healthcare team is paramount to ensure patients at risk receive preventive interventions for BCRAL, regardless of where they are receiving care.

**Funding:**

This study was not supported by any funding. 10.13039/501100009753RJC received investigator grant support from the 10.13039/501100000925Australian National Health and Medical Research Council (APP1194051).

**Research in context**

**Evidence before this study**

Using expert knowledge and data from the literature via PubMed and Embase between January 2018 and December 2022, we noted that there is an increasing amount of high-level evidence published on interventions to prevent breast cancer-related arm lymphoedema (BCRAL). However, some of these interventions, such as prospective surveillance programs, prophylactic compression sleeves and advanced surgical techniques, may significantly add workload to healthcare teams and impact healthcare resources. There is also heterogeneity in how these measures are implemented in the literature. Healthcare professionals may be uncertain about how to incorporate these strategies into their clinical practice, particularly in settings with stringent resources.

**Added value of this study**

This international Delphi study led by the Multinational Association in Supportive Care in Cancer (MASCC) Oncodermatology and Survivorship study groups provides consensus guidance amongst multidisciplinary experts regarding interventions to prevent BCRAL based on recently published high-level evidence. Specifically, this clinical practice guidance offers suggestions on how to implement these measures when there are resource constraints.

**Implications of all the available evidence**

This Delphi consensus study offers a guide for healthcare professionals and administrators on how to incorporate the latest level I evidence for the prevention of BCRAL. Future research needs to be performed on patient preferences for these interventions and the cost-effectiveness of these strategies.

## Introduction

Breast cancer-related arm lymphoedema (BCRAL) is a common complication experienced in up to one in five breast cancer patients after anti-cancer treatments.^1^ About 50% of patients with BCRAL develop this condition 12–30 months after surgery, but it can also develop many years later in the survivorship phase.^2^, ^3^, ^4^ As chronic lymphoedema is irreversible, difficult to treat and significantly affects patients’ health-related quality of life (HRQoL),^5^ prophylactic management to prevent lymphoedema, detection of early lymphoedema and halting its progression are important.^6^

Prospective surveillance programs have been developed and tested in randomised controlled trials (RCTs) to facilitate detection and intervention of early BCRAL.^7^^,^^8^ A systematic review and meta-analysis demonstrated that such programs were successful in reducing the incidence of chronic BCRAL.^9^ However, significant heterogeneity exists in the surveillance interval, total surveillance duration, methods of detecting early lymphoedema, and interventions that follow the diagnosis of early lymphoedema amongst studies.^9^ Recently, a RCT of 307 patients demonstrated that prophylactic compression sleeves applied after surgery were effective in delaying and reducing the incidence of arm swelling in patients who had axillary lymph node dissection (ALND).^10^ Prophylactic lymphatic reconstruction and axillary reverse mapping were also shown to be effective in preventing BCRAL in systematic reviews.^11^^,^^12^

A recent international Delphi consensus (2022) led by Martinez-Jaimez et al. included recommendations on surgical and radiation techniques, physiotherapy, exercise and dietary recommendations for the prevention of BCRAL, while helpful, it did not cover details of how prospective surveillance programs should be implemented and whether prophylactic sleeves are recommended.^13^ Characteristics of patients who should undergo prophylactic lymphatic reconstruction and axillary reverse mapping were also not defined. Healthcare professionals involved in the care of patients with breast cancer and lymphoedema may be uncertain whether these interventions should be adopted and how they should be implemented in their practice, especially when there are resource constraints.^14^

Given the publications of the most recent, emerging evidence, the Multinational Association in Supportive Care in Cancer (MASCC) Oncodermatology and Survivorship study groups formed a steering committee to generate a clinical practice guide on how to implement evidence-based interventions through a modified Delphi consensus process.

## Methods

### Objective

The objective of this modified Delphi consensus process was to develop recommendations on evidence-based interventions to prevent BCRAL when there are resource limitations.

### Steering committee

A steering committee consisting of 11 members was convened. Ten members were MASCC Oncodermatology and/or Survivorship study group members (HCYW, MPW, ACWC, ND, PB, JRW, CvdH, MF, EC, and RJC). A lymphoedema patient advocate (MB) was invited to the committee to provide the patient perspective on survey development. All committee members were involved in the literature search, survey planning, development, data analysis, interpretation of findings and approval of the final statements.

### Literature search

As part of survey development, a literature search was performed in December 2022 by the steering committee using keywords “prevention” and “breast cancer-related arm lymphoedema” in PubMed and EMBASE to identify published research articles related to the prevention of BCRAL from 2018 to 2022. Only RCTs and the most updated systematic reviews (SRs) on interventions that were deemed significant to clinical practice and that have impact on resource allocation in healthcare systems were selected as the basis of the Delphi study by the steering committee. As patients may be triaged to receive prevention strategies based on the risk of developing BCRAL in resource constrained settings, a search of the databases and grey literature were performed to identify updated SRs on risk factors of BCRAL. The steering committee identified six articles that formed the basis of the Delphi study: (1) SR on risk factors of BCRAL, (2) SR on prospective surveillance programs for BCRAL, (3) RCT on prophylactic compression sleeves, (4) RCT on axillary radiotherapy versus ALND in patients with a positive sentinel lymph node (5) SR on prophylactic lymphatic reconstruction, and (6) SR on axillary reverse mapping.^9^, ^10^, ^11^, ^12^^,^^15^^,^^16^ The key findings of the six papers are summarised in Table 1.

**Table 1:** Papers that the international Delphi consensus is based on.

Author	Title of study	Study type	Key findings
Shen et al.^15^	Risk factors of unilateral breast cancer-related lymphedema: an updated systematic review and meta-analysis of 84 cohort studies	SR	From 84 cohort studies involving 58,538 patients, 14 risk factors of BCRAL were identified.
Rafn et al.^9^	Prospective surveillance and targeted physiotherapy for arm morbidity after breast cancer surgery: a pilot randomized controlled trial	SR	Based on 21 studies evaluating BCRAL, patient participation in prospective surveillance with early management reduced risk of chronic BCRAL compared to usual care.
Paramanandam et al.^10^	Prophylactic Use of Compression Sleeves Reduces the Incidence of Arm Swelling in Women at High Risk of Breast Cancer–Related Lymphedema: A Randomized Controlled Trial.	RCT	In this RCT involving 307 patients at high risk of lymphoedema, prophylactic use of compression sleeves reduced incidence of post-operative arm swelling in the first year after breast cancer surgery without impacting quality of life.
Bartels et al.^16^	Radiotherapy or Surgery of the Axilla After a Positive Sentinel Node in Breast Cancer: 10-Year Results of the Randomized Controlled EORTC 10981–22023 AMAROS Trial	RCT	In this RCT involving 4806 patients, axillary radiation was non inferior to axillary lymph node dissection in terms of 10-year axillary recurrence rates in cT1-2 breast cancer patients with a positive sentinel lymph node biopsy. Patients in the axillary radiation arm had significantly lower rates of BCRAL.
Cook et al.^12^	Immediate Lymphatic Reconstruction to Prevent Breast Cancer-Related Lymphedema: A Systematic Review	SR	Based on 5 studies involving 251 patients, immediate lymphatic reconstruction reduced the risk of BCRAL from 30.5% to 6.6% at a median follow-up of 22.6 months.
Co et al.^11^	Axillary Reverse Mapping in the Prevention of Lymphoedema: A Systematic Review and Pooled Analysis	SR	Based on 5 RCTs with 1696 patients, axillary reverse mapping reduced the incidence of BCRAL from 18.8% to 4.8%, without increasing the axillary recurrence rate at a median follow-up of 37 months.

Abbreviations: BCRAL, breast cancer-related arm lymphoedema; SR, systematic review; RCT, randomised controlled trial.

### Delphi consensus process

A two round modified Delphi consensus process was conducted over a period of 3 months (February 27, 2023–May 25, 2023). Surveys were developed through the Qualtrics web-based development tool. The responses given by individual participants were anonymized to protect participant confidentiality. This study did not involve any patients as participants. Only participants who took part in the first round were invited to participate in the second round.

Members of the MASCC Oncodermatology and Survivorship Study Groups were invited to participate in the Delphi process if they had an interest in lymphoedema, as shown by previously published research in the area or expressed an interest during MASCC Study Group meetings. Corresponding authors of the six original articles that the Delphi study was based on and the studies with the five largest number of patients included in each of the SRs were invited. Additionally, executive members of international lymphoedema societies were invited to represent a broad range of experts on lymphoedema worldwide. Local experts with extensive experience in breast cancer or lymphoedema care identified by the steering group were also invited. Using the snowball method, experts were invited to refer colleagues who were eligible to participate in the survey. Emails were sent to all potential participants by the steering committee to ask for their interest to participate. Both MASCC and non-MASCC members were eligible to be a participant.

Following informed consent, participants were directed to an online survey to complete. In the first round, participants were provided a summary of the evidence that correspond to each of the statements in the survey (Supplementary Information A—Evidence Summary Sheet), and links to the six original articles for reference. Participants were invited to rate 12 risk factors of BCRAL based on their level of importance (“high”, “intermediate” and “low”) and select the most important 5 risk factors. Participants were then asked about the extent of agreement or disagreement on a 5-item Likert scale (“completely agree”, “agree”, “neutral”, “disagree”, and “completely disagree”) on 46 statements developed by the investigating team based on the six articles. Respondents who “disagree” or “strongly disagree” with statements would be asked to provide reasons why they selected this choice in a free-text field within the survey (Supplementary Information B—Round 1 Survey). As per published recommendations, statements that reached at least 75% of participants voting “completely agree” or “agree” were deemed to have achieved consensus.^17^

After participant voting, the steering committee compiled and prepared the results from the first round. The steering committee refined the answer choices for the second round after several expert panel members commented that some sections of the survey was beyond their scope of expertise. The “neutral” option was amended to “do not know or not within my scope of practice to judge” to better reflect the opinions of these participants. Statements that did not reach consensus were reviewed and amended based on participant feedback by the steering committee. For the second round, statements that did not reach consensus or were newly created/modified based on participant feedback were sent as a survey to the same participants (Supplementary Information C—Round 2 Survey). Participants were shown the results of the first round and where amendments were made to the statements in the second round. In the study protocol, a third round (or subsequent rounds) was planned if consensus was not achieved in statements in the second round with the same methodology, but this was not the case for our study as all statements achieved consensus in the second round.

### Ethics

This study was prospectively approved by the Flinders University Human Ethics Low Risk Panel (Project Number: 5937). All participants of the expert panel provided informed consent.

### Consensus statement disclaimer

The recommendations provided in this publication reflect the majority opinion of experts of the expert panel. Although the recommendations are meant to guide clinical decision-making, they should not be considered as inclusive of every possible method to prevent breast cancer-related lymphoedema. With novel evidence constantly emerging, these recommendations might not be reflective of all the latest evidence, but only those that have significant impact on healthcare resources as determined by the steering group. These consensus-based recommendations are intended to provide guidance for general practitioners, dermatologists, radiation oncologists, medical oncologists, surgeons, nurses, physiotherapists, occupational therapists and rehabilitation physicians. However, the treatment decision for each patient should ultimately be made at the discretion of the treating clinician in conjunction with the patients’ unique needs and shared decision-making. This clinical practice guidance only focuses on BCRAL and is not applicable to arm lymphoedema caused by other primary cancers or lymphoedema of other body sites.

### Role of the funding source

There was no funding source for this study.

## Results

Two Delphi consensus rounds were completed. Among 102 invited experts, 64 experts consented to participate in the first round, of which 55 completed the second round and were included as members of the expert panel (Appendix 1). The age and gender identity of the participants from the first round were outlined in Table 2. A majority of participants identified as female (60.9%) and belonged to the age range of 35–44 years old (39.1%). The response rate for the second round was 86%. Among the expert panel, 16 countries or regions were represented, including Japan (n = 10), USA (n = 9), Hong Kong (n = 9), Canada (n = 6), Italy (n = 4), Denmark (n = 3) and others (n = 14) (Table 3).

**Table 2:** Age range and gender identity of participants from the first round.

Age	
Age range	Number of participants (%)
18–24	1 (1.6%)
25–34	4 (6.3%)
35–44	25 (39.1%)
45–54	14 (21.9%)
55–64	13 (20.3%)
65–74	7 (10.9%)
**Gender**	
Female	39 (60.9%)
Male	24 (37.5%)
Non-binary/Third Gender	1 (1.6%)

**Table 3:** Demographics of the MASCC BCRAL expert panel.

Countries represented		Disciplines represented	
Japan	10	Radiation Oncologist	18
USA	9	Physiotherapist	8
Hong Kong	9	Surgeon	7
Canada	6	Clinical Oncologist	6
Italy	4	Researcher	4
Denmark	3	Oncology Nurse	3
Australia	2	General Practitioner/Family Physician	2
Belgium	2	Occupational Therapist	2
Spain	2	Rehabilitation physician	2
United Kingdom	2	Massage Therapist	1
Brazil	1	Lymphoedema Therapist	1
India	1	Dermatologist	1
Mexico	1		
Poland	1		
Singapore	1		
Turkey	1		

The panel comprised of radiation oncologists (n = 17), physiotherapists (n = 8), surgeons (n = 7), clinical oncologists (physicians trained in both radiation oncology and medical oncology) (n = 7), researchers in lymphoedema (n = 4), oncology nurses (n = 3) occupational therapists (n = 2), rehabilitation physicians (n = 2), general practitioners (n = 2), a dermatologist (n = 1), a lymphoedema specialist (n = 1) and a massage therapist (n = 1) (Table 3). Over 75% of the expert panel had experience in treating or conducting research in patients with breast cancer or BCRAL for at least 10 years.

**Fig. 1:**
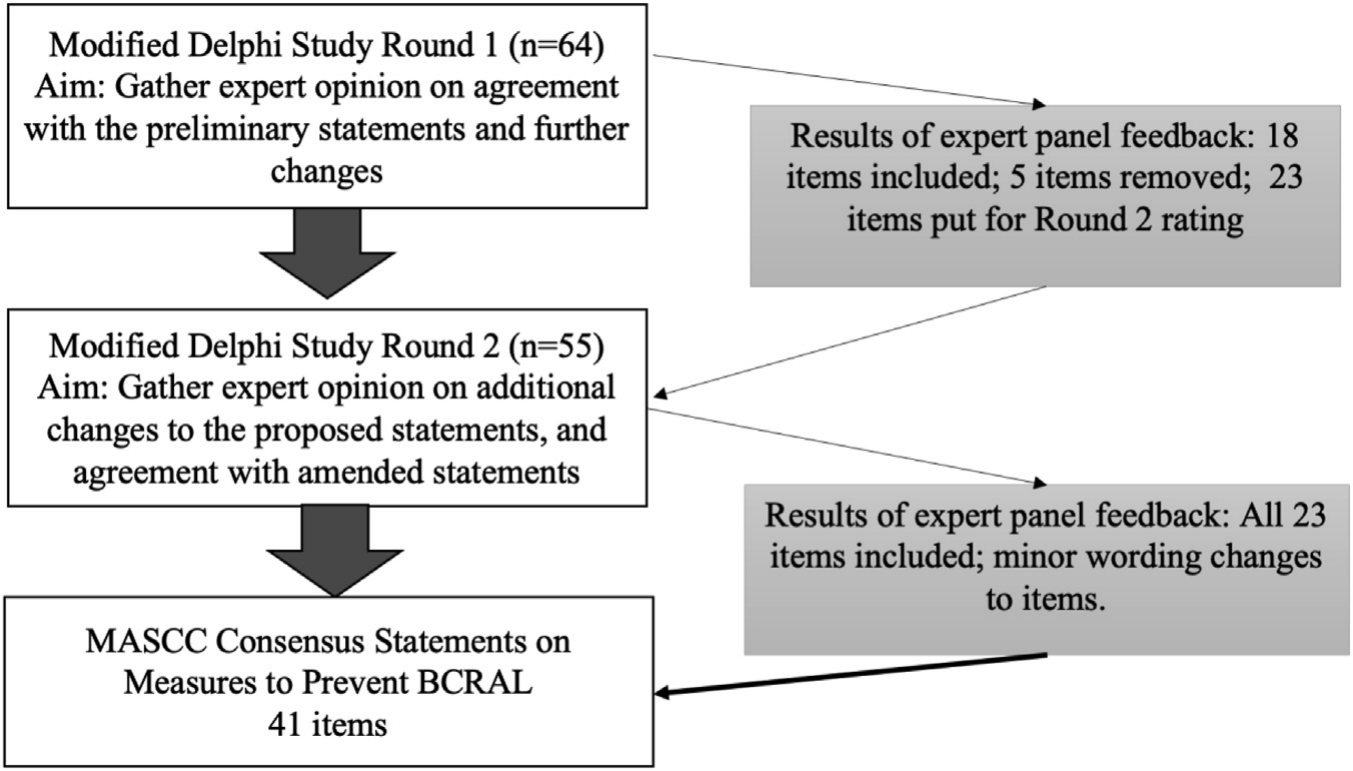
**Modified Delphi consensus process**. Abbreviations: n = number of participants.

Out of 46 statements, consensus was achieved in 18 in the first round. After reviewing participant feedback, the steering committee decided to remove 5 statements and combine them with other statements that did not reach consensus for re-rating. There were 23 statements sent back to the expert panel for rating in the second round. All of them achieved consensus in the second round (Fig. 1). Individual statements that reached consensus are summarised below and listed in Table 4.

**Table 4:** Final consensus statements after a two-round Delphi consensus process.

Statement number	Final statement	Round that the statement achieved consensus and percentage of experts that agreed
**Risk factors for breast cancer related arm lymphoedema**		
1.1	When there are resource constraints, patients who present with a higher BMI (BMI ≥30 kg/m2) should be prioritised over patients with BMI <30 kg/m2 when selecting patients for prophylactic management of lymphoedema.	Consensus achieved in round 1 76.6% (49/64)
1.2	The timing of chemotherapy (neoadjuvant versus adjuvant) may impact the subsequent risks of lymphoedema but should not be a major determining factor on selecting patients for prophylactic management of lymphoedema until more studies are available.	Consensus achieved in round 2 92.5% (49/53)
1.3	The type of chemotherapy (taxane versus non-taxane) a patient receives may impact the subsequent risks of lymphoedema, but should not be a major determining factor on selecting patients for prophylactic management of lymphoedema until more studies are available.	Consensus achieved in round 2 83% (44/53)
1.4	When there are resource constraints, patients who had ≥15 axillary lymph nodes removed in axillary dissection should be prioritised over patients who had less lymph nodes removed when selecting patients for prophylactic management of lymphoedema.	Consensus achieved in round 1 84.4% (54/64)
1.5	When there are resource constraints, patients who received axillary radiation should be prioritised over patients who receive radiation to the breast/chest wall ± the supraclavicular fossa when selecting patients for prophylactic management of lymphoedema.	Consensus achieved in round 1 90.6% (58/64)
**Prospective surveillance programs**		
2.1	A prospective surveillance program is recommended to reduce risks of chronic lymphoedema after breast cancer surgery where feasible and resources allow	Consensus achieved in round 1 93.8% (60/64)
2.2	Bioimpedance spectroscopy is one of the more commonly used methods in the literature for early lymphoedema detection in prospective surveillance programs and can be an option before more prospective studies are available to suggest the preferred method of assessment.	Consensus achieved in round 2 89.8% (44/49)
2.3	In a prospective surveillance program, arm circumference (or volumetric) or lymphangiography/lymphoscintigraphy measurement is an alternative method to identify patients with subclinical/early stage lymphoedema for early treatment when bioimpedance spectroscopy is not available or there are resource limitations	Consensus achieved in round 1 82.8% (53/64)
2.4	In a prospective surveillance program, treatment is triggered when the bioimpedance spectroscopy score shows an increase of L-Dex ≥6.5 compared to pre-surgical values	Consensus achieved in round 2 91.9% (34/37)
2.5	In a prospective surveillance program, treatment can be triggered when a difference in volume measurements of ≥5 but <10% is seen compared to pre- surgery values	Consensus achieved in round 2 88.6% (39/44)
2.6	In a prospective surveillance program, treatment should be triggered by any patient- reported arm symptoms (e.g., swelling, heaviness, tightness, and numbness)	Consensus achieved in round 1 81.3% (52/64)
2.7	In a prospective surveillance program, more intensive treatment is indicated (e.g., complete decongestive therapy) when a difference in bioimpedance spectroscopy scores is L-Dex >10 compared to pre-surgery values	Consensus achieved in round 2 94.9% (37/39)
2.8	In a prospective surveillance program, more intensive treatment (e.g., congestive decompressive therapy) is indicated when a difference in volume measurements is ≥ 10% compared to pre-surgery values	Consensus achieved in round 2 91.7% (44/48)
2.9	In a prospective surveillance program, the diagnosis of chronic lymphoedema is made when there are persistent symptoms despite initial treatments	Consensus achieved in round 1 75.0% (48/64)
2.10	Prospective surveillance is recommended to start within 3 months after surgery	Consensus achieved in round 1 82.8% (53/64)
2.11	Pre-surgical assessment of lymphoedema is required in a prospective surveillance program for better comparison of measurements after surgery	Consensus achieved in round 1 89.1% (57/64)
2.12	The surveillance interval for a prospective surveillance program is recommended to be every 3–4 months in the first year then every 6–12 months thereafter where feasible and resources allow	Consensus achieved in round 1 85.9% (55/64)
2.13	The total duration of surveillance in a prospective surveillance program is recommended to be at least 24 months from surgery where feasible and resources allow	Consensus achieved in round 1 89.1% (57/64)
2.14	In a prospective surveillance program, healthcare professionals should conduct the prospective surveillance where feasible and resources allow	Consensus achieved in round 1 95.3% (61/64)
2.15	In a prospective surveillance program, patients or family members who receive adequate training should conduct the prospective surveillance where resources are limited	Consensus achieved in round 1 81.3% (52/64)
2.16	When subclinical/early stage lymphoedema is detected in a prospective surveillance program, compression garment are recommended for initial treatment	Consensus achieved in round 1 76.6% (49/64)
2.17	When subclinical/early stage lymphoedema is detected in a prospective surveillance program, compression sleeves are suggested to be prescribed for at least 4–6 weeks. A longer duration can be considered depending on clinical response and the individual judgement of the treating therapist.	Consensus achieved in round 2 96.2% (50/52)
**Prophylactic compression sleeves**		
3.1	Prophylactic compression sleeves should be offered as an option to prevent breast cancer- related arm lymphoedema	Consensus achieved in round 2 80% (40/50)
3.2	For patients at high risk of lymphedema who wish to consider prophylactic arm sleeves, the sleeves should be applied from the first post- operative day until 3 months after the completion of adjuvant treatments (excluding hormonal treatments)	Consensus achieved in round 2 87.8% (43/49)
3.3	The daily use of prophylactic arm sleeves is suggested to be at least 8 h. The duration should be individualized depending on patients' preferences and comfort while wearing the sleeves.	Consensus achieved in round 2 92.5% (49/53)
3.4	The pressure of the prophylactic arm sleeve should be reviewed regularly and adjusted to patients' risks and needs.	Consensus achieved in round 2 96.3% (52/54)
3.5	Patients should be assessed every 6 months for any lymphoedema while using prophylactic compression sleeves	Consensus achieved in round 1 76.6% (49/64)
3.6	Patients should be assessed for breast cancer related arm lymphoedema using bioimpedance testing while using prophylactic compression sleeves	Consensus achieved in round 1 75% (48/64)
3.7	Patients should be assessed for breast cancer related arm lymphoedema using relative volume measurements while using prophylactic compression sleeves	Consensus achieved in round 1 85.9% (55/64)
3.8	When prophylactic compression sleeves are used, clinical lymphoedema diagnosed by bioimpedance testing or increase in relative arm volume by ≥ 10% should trigger subsequent treatments	Consensus achieved in round 1 85.9% (55/64)
**Axillary radiation instead of axillary lymph node dissection for positive sentinel lymph node biopsy**		
4.1	Axillary lymph node dissection should not be routinely offered to breast cancer patients with clinical T1 or T2, node-negative disease who are found to have 1 to 2 positive sentinel lymph nodes and received breast conservation therapy	Consensus achieved in round 2 97.6% (40/41)
4.2	Axillary radiotherapy instead of axillary lymph node dissection can be considered in breast cancer patients with clinical T1 or T2, node-negative disease who are found to have 1 to 2 positive sentinel lymph nodes and received mastectomy	Consensus achieved in round 2 92.3% (36/39)
4.3	Axillary lymph node dissection should be offered instead of axillary radiation in breast cancer patients with clinical T1 or T2, node-negative disease who are found to have more than 2 positive sentinel lymph nodes	Consensus achieved in round 2 84.2% (32/38)
4.4	For clinical T1 or T2, node- negative breast cancer patients with high risk tumour biology (e.g., triple negative, grade 3) who are found to have 1 to 2 positive sentinel lymph nodes, an individualized decision should be made with the patient whether to perform axillary dissection or give axillary radiation.	Consensus achieved in round 2 95% (38/40)
4.5	In clinical T1 or T2, node- negative disease who are found to have positive sentinel lymph nodes with less than 2 lymph nodes removed or having extra- nodal extension, an individualized decision should be made with the patient whether to perform axillary dissection or give axillary radiation.	Consensus achieved in round 2 97.6% (41/42)
4.6	When axillary radiation is recommended in clinical T1-2 node negative breast cancer with a positive sentinel lymph node, the decision to include internal mammary chain in the radiation volumes should be individualized depending on factors such as location of primary tumour and presence of cardiac risk factors	Consensus achieved in round 2 100.0% (39/39)
4.7	While there are concerns that axillary radiotherapy is associated with a relatively higher incidence of second primary cancers, the decision to offer radiotherapy should not be affected if indicated.	Consensus achieved in round 2 90.0% (36/40)
4.8	In accordance with international guidelines, moderate hypofractionation (40–42.5 Gy in 15–16 fractions) is preferred over 50 Gy in 25 fractions when axillary radiotherapy is given to clinical T1 or T2, node-negative patients with a positive sentinel lymph node biopsy	Consensus achieved in round 2 97.3% (36/37)
**Prophylactic lymphatic reconstruction**		
5.1	Where expertise is available and resources allow, prophylactic lymphatic reconstruction is an option to reduce risks of chronic breast cancer related lymphoedema in patients who require extensive axillary lymph node dissection for large or multiple clinically positive lymph nodes.	Consensus achieved in round 2 92.7% (38/41)
**Axillary reverse mapping**		
6.1	Where expertise is available and resources allow, axillary reverse mapping is an option for patients indicated for axillary lymph node dissection to reduce risks of chronic breast cancer related lymphoedema.	Consensus achieved in round 2 97.9% (46/47)
6.2	Axillary reverse mapping should not be offered in patients at high risk of axillary recurrence (e.g., multiple clinically positive lymph nodes, T4 primary).	Consensus achieved in round 2 94.1% (32/34)

### Risk factors of BCRAL

In the first round, risk factors that were ranked by more than 50% of experts as the top five most important were: ALND (90.6%), use of post-operative radiotherapy (84.4%), relative within-arm volume increase one month after surgery (65.6%), greater number of lymph nodes dissected (60.9%) and higher body mass index (BMI) (59.4%) (Table 5). Risk factors that were rated as having “high importance” were ALND (96.9%), greater number of lymph nodes dissected (76.6%) and relative within-arm volume increase one month after surgery (71.4%).

**Table 5:** Risk factors of breast cancer related arm lymphoedema listed according to percentage of expert panel members selecting them as a top five risk factor.

Risk factor	Number of participants selecting factor as a top five risk factor (out of 64)	Percentage of participants selecting factor as a top five risk factor
Axillary lymph node dissection	58	90.6%
Use of postoperative radiotherapy	54	84.4%
Relative arm volume increase	42	65.6%
Number of lymph nodes dissected	39	60.9%
Body mass index	38	59.4%
Post-operative complications	32	50.0%
Use of chemotherapy	13	20.3%
Tumour size	12	18.8%
Mastectomy	11	17.2%
Presence of hypertension	9	14.1%
Cancer stage	8	12.5%
Ethnicity	4	6.3%

When there are resource limitations, the expert panel recommended prioritizing interventions to prevent BCRAL for patients who have BMI greater or equal to 30 kg/m^2^, more than or equal to 15 axillary lymph nodes dissected, and patients who received axillary radiotherapy (compared to patients who received radiation to the chest wall or breast with or without supraclavicular fossa). The timing of chemotherapy (neoadjuvant versus adjuvant) and choice of chemotherapy received (taxane versus non-taxane) should not be major determining factors that affect the decision of healthcare professionals to offer prophylactic management of BCRAL until more studies are available.

### Prospective surveillance programs

The expert panel recommended that prospective surveillance should be implemented where feasible and resources allow. Bioimpedance spectroscopy (BIS) was recommended as an option for early lymphoedema detection before more prospective studies are performed to suggest the preferred method of detection. Other methods, such as arm circumference, volumetric measurements, lymphangiography and lymphoscintigraphy were alternatives to BIS when it is not available or there are resource limitations (see remarks in Discussion).

Thresholds to trigger early intervention in a surveillance program recommended by the expert panel were: (1) when an increase in L-Dex score is ≥6.5 in BIS compared to pre-surgical values (2) when a difference in volume measurements of ≥5 but <10% is seen compared to pre-surgery values and (3) when the patient has any arm symptoms such as swelling, heaviness, tightness and/or numbness.

When early lymphoedema is detected in a surveillance program, the panel recommended compression sleeves as the initial treatment for at least 4–6 weeks. A longer duration can be recommended depending on clinical response and the individual judgement of the treating therapist.

When patients are found to have L-Dex >10 or volume measurements are ≥10% compared to pre-surgery values or persistent symptoms despite initial treatments, more intensive treatment (e.g., complete decongestive therapy) is indicated.

The panel also made recommendations on the schedule and logistics of a prospective surveillance program. First, pre-surgical measurements should be performed to allow better comparisons after surgery. The program should start within 3 months after surgery, and assessments repeated every 3–4 months in the first year then every 6–12 months thereafter for at least 24 months from surgery where feasible and resources allow. Surveillance should be performed by trained healthcare professionals, but patients or family members who receive adequate training are recommended to conduct the surveillance if resources are limited.

### Prophylactic compression sleeves

The expert panel recommend that prophylactic compression sleeves should be offered as an option to patients at risk of BCRAL (see remarks in Discussion). The sleeves should be applied from the first day after surgery to 3 months after the completion of adjuvant treatments (excluding hormonal treatments).

The daily use of the sleeve is recommended to be at least 8 h, but should be individualised to patients' preferences and comfort while wearing the sleeves. The pressure of the sleeve should be regularly reviewed and adjusted according to patients’ risks and needs.

While being treated on the prophylactic sleeves, the expert panel recommended that patients are assessed by BIS and relative volume measurements every 6 months after the start of treatment. Clinical lymphoedema diagnosed by BIS or an increase in relative arm volume by ≥10% should trigger subsequent treatments.

### Axillary management after a positive sentinel lymph node biopsy

The expert panel recommended that routine ALND should not be offered in patients with clinical T1–2, node negative disease, with 1–2 positive sentinel lymph nodes (SLN) who receive breast conservation therapy (BCT). In the same group of patients who received mastectomy, axillary radiation can be considered instead of ALND. When patients are found to have more than two positive SLNs, the panel recommended that ALND should be performed.

An individualised decision of axillary radiation versus ALND should be made when patients have less than 2 SLNs biopsied, the positive lymph node is shown to have extra-nodal extension or when patients have high risk tumour biology such as grade 3 or triple negative disease.

When prescribing axillary radiation, the panel recommended offering moderate hypofractionation (40–42.5 Gy in 15–16 fractions) according to international guidelines. The decision to include the internal mammary chain in the radiation volumes should be individualised based on the presence of cardiac risk factors and the location of the tumour. While there are concerns that axillary radiotherapy is associated with a relatively higher incidence of second primary cancers, the decision to offer radiotherapy should not be affected if indicated (see remarks in Discussion).

### Prophylactic lymphatic reconstruction

Prophylactic lymphatic reconstruction was recommended by the expert panel as an option to prevent BCRAL where the expertise is available and resources allow in patients who require extensive ALND for large, multiple lymph nodes.

### Axillary reverse mapping

The panel recommended that axillary reverse mapping is an option to prevent BCRAL for patients indicated for ALND where the expertise is available and resources allow. However, patients at high risk of axillary recurrence, for example multiple positive axillary lymph nodes or T4 primary, are not recommended to receive this treatment.

## Discussion

This international Delphi consensus provides recommendations on interventions to prevent BCRAL. The steering committee specifically selected interventions from recently published, high-impact articles that are potentially practice-changing and could impact healthcare resources as the basis of this Delphi study. The consensus statements generated from this study and approved by the participant panel provide a timely practical guide for healthcare professionals and administrators who are looking to incorporate evidence-based interventions to prevent BCRAL in their institution.

Compared to the international Delphi consensus led by Martinez-Jaimez et al.,^13^ all statements of our study were based on SRs and RCTs. Their team did not specify which study designs their statements were based on in their methods. The advantage of our approach is that experts could refer directly to the data that the statement was referenced upon while completing the survey. The expert panel could rate statements based on their critical assessment of the evidence in addition to their research and clinical experience. The Delphi study of Martinez-Jaimez et al. should be regarded as a complimentary guide to ours when the healthcare team is seeking practical recommendations from evidence that extends beyond large RCTs or SRs, such as skin care advice, guidance on dietary supplements or the types of physical exercise (and modifications) to suggest to patients.

To our knowledge, there are no detailed guidelines currently available to recommend how prospective surveillance programs should be implemented, although this approach has been advocated by both the National Comprehensive Cancer Network (NCCN) and International Society of Lymphology (ISL) guidelines.^18^^,^^19^ This Delphi study is the first to provide recommendations on the frequency and duration of surveillance, methods to detect early lymphoedema, trigger thresholds for early intervention, and types of treatments to offer depending on the degree of lymphoedema. Going forward, the preparedness of healthcare teams to implement these programs should be assessed in the real world. Prospective surveillance programs will require additional resources to perform regular assessments on patients. The MASCC Survivorship and Oncodermatology groups will be leading an international healthcare professional survey to understand how surveillance programs are currently implemented in different resource settings and evaluate the barriers of implementation. More research will be required to evaluate the efficacy of self-managed, remotely administered and multi-modal programs in maximising availability of surveillance to all patients at risk at lower costs.

Even with prospective surveillance programs in place, patients may develop BCRAL in between the fixed surveillance intervals. Educating patients about the signs and symptoms of BCRAL are highly important to ensure patients know when to seek early medical attention. In the RCT by Shi et al., patients who received an education program on BCRAL had better arm function and quality of life compared to those who did not. The incidence of BCRAL at four months was also lower, although the difference was not statistically significant (3.6% versus 7.1%, p = 0.744).^20^ It is possible that the difference could become significant if patients were followed up for a longer period of time. This study highlighted that empowering patients with knowledge about BCRAL is a powerful method to improve patient outcomes. However, healthcare professionals need to ensure that the information relayed to patients in education programs is evidence based. For example, the 2012 National Lymphedema Network Position Statement advocated precautionary measures such as avoiding blood pressure measurements on the at-risk limb and wearing a compression sleeve during air travel to reduce the risk of BCRAL.^21^ Many of these suggestions were based on expert opinion and physiological principles. Subsequent large cohort studies published after the position statement showed that there were no correlation between these risk factors and the development of BCRAL.^22^, ^23^, ^24^ Patients may have a false sense of security after adhering to these lifestyle modifications if the recommendations are not supported by evidence.

Our Delphi consensus presented recommendations on the methods of detecting early BCRAL and thresholds to initiate treatment with compression sleeves in a surveillance program. We recognise that there is a significant heterogeneity in the methods of detection, thresholds for the respective methods and follow-up treatments (e.g., compression sleeves, congestive decompressive therapy, surgery) reported in the literature.^9^ BIS was one of the most frequently used methods of detection in the studies on surveillance programs.^9^ While assessments using BIS is non-invasive and quick, whether this method is more superior compared to other less expensive methods (e.g., perometry and tape measurements) in detecting early BCRAL warrants further study. In the PREVENT trial, patients were randomised to receive BCRAL surveillance by BIS or traditional tape measurement. At a median follow up of 32.9 months, interventions were triggered at a significantly lower rate in the BIS arm compared to the tape measurement arm (20.1% versus 27.5%, p = 0.001). The rates of progression of BCRAL in those who received interventions were lower in the BIS arm (7.9% versus 19.2% p = 0.016).^25^ Authors of the PREVENT trial stated that BIS was useful in identifying a group of patients who better benefit from early compression. Brunelle et al. questioned this conclusion as there was a lower rate of trigger in the BIS arm without any prior intervention, while previous studies reported that BIS tended to overestimate the incidence of BCRAL compared to other methods such as tape measurements and perometry.^10^^,^^26^^,^^27^ The lower rate of progression of BCRAL observed in the BIS arm could have been contributed by an imbalance of risk factors of BCRAL development in favour of the BIS arm. In the PREVENT trial, the BIS arm had more patients with stage I disease and less ALND performed compared to the tape measurement arm.^25^ Taking the above points into consideration, BIS should be considered an option for BCRAL screening, but not a standard of care following the publication of the PREVENT trial. Healthcare teams should select a detection method based on the cost, availability of the tool and experience of using it. Practitioners should remain flexible and carefully discuss with patients the pros and cons of initiating interventions when thresholds are met, taking into account whether patient has any symptoms and risk factors of BCRAL. With studies showing that the level of agreement across different tools are limited,^10^^,^^27^, ^28^, ^29^ patients with early BCRAL should be monitored with the same detection methods to assess whether the intervention is useful. It should also be highlighted that the studies validating these thresholds were predominantly performed in patients from North American and European countries.^30^, ^31^, ^32^, ^33^, ^34^ Further work is needed to align thresholds for patients of different racial backgrounds in order of generate accurate incidence and prevalence estimates in future clinical studies. Institutions adopting the thresholds recommended in this paper should regularly review how they reflect their local populations and make adjustments accordingly.

The application of compression sleeves was recommended by our expert panel as the treatment of choice when early lymphoedema is detected in a prospective surveillance program. This recommendation is supported by the recently published RCT by Johansson et al., which randomized patients to receive a compression sleeve versus observation after early BCRAL was diagnosed (defined as relative arm volume increase of ≥5 to ≤8%).^35^ This trial showed that a significantly less proportion of patients in the compression sleeve group had progression into chronic BCRAL compared to observation group at 12 months (67% versus 31%, p = 0.012). On the contrary, another recently published RCT, the PLACE trial, failed to show that compression sleeves was useful in preventing progression of BCRAL after a 2-year follow up.^36^ A possible explanation is that more than 60% of patients in the PLACE trial were overweight (BMI ≥25 kg/m^2^) and experienced weight gain during the study period, which could have reduced the efficacy of the sleeves. Further research is needed to test whether more intensive strategies, such as the combination of exercise programs and compression sleeves, will be helpful to reduce the risk of progression of BCRAL in patients with high BMI.

This expert panel recommended that prophylactic compression sleeves should be offered to patients as an option for the prevention of BCRAL. Consensus was achieved in the second round when the statement was amended that this intervention should be offered as an “option”. Experts who disagreed reflected that the results from a single RCT should not be generalised for routine practice.^10^ Panel members also commented that the follow-up time of one year in the same study was not adequate to reflect its longer-term efficacy for preventing BCRAL. Healthcare professionals offering compression sleeves should counsel patients that the benefit of the intervention in preventing BCRAL that develops beyond one year is uncertain. This point should be especially emphasized to patients who receive both ALND and regional lymph node irradiation, as the peak to development of BCRAL in this group of patients is 18–24 months.^2^ Future RCTs should be performed with a longer follow-up to confirm the conclusions of the study. Nevertheless, compression sleeves are relatively low cost, associated with minimal risk and readily available. As some patients can develop BCRAL as early as within the first 6 months after surgery,^4^ prophylactic compression sleeves is a reasonable option for patients who wish to minimise the risk of early BCRAL. Real world, pragmatic studies are needed to understand patient uptake in the use of sleeves and whether there are any issues with sleeve fitness that may compromise efficacy, as these practical aspects were not reported in the RCT.^10^ Studies are also needed to test whether different types of sleeves (e.g., flat knit sleeves instead of circular-knit sleeves that was used in the trial) or adjusting the schedule of wearing the sleeves could further enhance their efficacy, as a proportion of patients still developed BCRAL in the experimental arm.

This Delphi study also offered practical recommendations for physicians of a multidisciplinary team on how to manage a positive SLN biopsy in early breast cancer. Minimizing long-term complications of treatment is especially crucial in this patient population because their prognosis is generally more favourable compared to patients with locally advanced disease. It is important to note that all statements in this section of the Delphi study did not reach a consensus in the first round. Consensus was achieved only after modifications of the statements based on the experts' qualitative feedback. An important reason was that the AMAROS study was performed between 2001 and 2010.^16^ Radiation techniques and dose schedules at that time were different compared to practice in the past 10 years. For example, patients in the AMAROS study were treated with a dose of 50 Gray in 25 daily fractions.^16^ However, hypofractionation has been increasingly used in the recent decade and recommended in international guidelines.^37^^,^^38^ Additionally, statements in the first round were designed to see whether experts would generalise the results of the AMAROS study to different patient characteristics. Many experts who disagreed in the first round commented that statements should also reflect the results of the Z0011 study, which reported that axillary recurrence rates of observation after SLN biopsy were non-inferior to that after ALND in patients receiving BCT.^39^ After modification of the statements to combine elements of the AMAROS and Z0011 studies, consensus was achieved in the second round. Although both studies included patients with more aggressive tumour biology (e.g., grade 3 or triple negative disease) and patients with less than 2 SLNs sampled,^16^^,^^39^ experts were concerned that there is a risk of under-treatment with axillary radiation alone. The decision for axillary radiation and ALND should thus be individualised based on patients’ risks and preferences in these cases.

In the AMAROS study, a statistically significant higher incidence of second primary cancers was observed in the axillary radiotherapy group compared to the axillary lymph node dissection group.^16^ Epidemiologic studies have shown that radiotherapy can be associated with second primary malignancies within or near the radiation fields.^40^ However, a majority of patients in the axillary lymph node dissection group also received adjuvant chest wall or breast radiation therapy. Therefore, the reason for this observation in the exploratory analysis of the AMAROS study may not be explained solely by a slightly larger radiation target volume in patients receiving axillary radiotherapy. Moreover, since the incidence of secondary cancers was not one of the endpoints of the study, the study was underpowered and not balanced for this type of analysis. Nevertheless, the authors of the AMAROS trial and a subsequent commentary by Esserman et al. suggested that we could neither confirm nor exclude the possibility of a relationship between axillary radiotherapy and second primary cancers based on the data.^41^ At the time when the Delphi consensus was carried out, the expert panel recognized the possible correlation between axillary radiotherapy and a higher incidence of second primary cancers, but given that the explanation for this is unclear, recommended that the decision to offer axillary radiation if indicated should not be affected by the exploratory analysis. A recent Early Breast Cancer Trialists' Collaborative Group (EBCTCG) individual patient data meta-analysis evaluating regional lymph node irradiation has solidly refuted the suggestion of the AMAROS study that axillary radiation is associated with a higher risk of second primary cancers, in particular contralateral breast cancers (personal communication from Dr Philip Poortmans on the results of “Radiotherapy to regional nodes in early breast cancer: individual-patient-data meta-analysis of 14,324 women in 16 trials”, accepted and pending publication in The Lancet).

The recommendations made by the expert panel regarding the management of a positive SLN biopsy were largely in concordance to that of the latest Ontario Health (Cancer Care Ontario [CCO]) and American Society of Clinical Oncology (ASCO) guideline on the management of axilla in early breast cancers.^42^ An additional recommendation from the CCO/ASCO guideline not mentioned by our expert panel was that omitting radiation therapy is an option for patients with small tumour size, favourable tumour features (e.g., oestrogen receptor positive patients undergoing hormonal therapy), clear margins, and 1–3 positive SLNs who are treated with chemotherapy or endocrine therapy. While omitting radiation therapy would further reduce the risk of BCRAL, the two RCTs that this recommendation was based on showed a higher risk of locoregional recurrence in patients who did not receive radiation therapy despite having the same overall survival as those given radiation therapy.^43^^,^^44^ Members of a multidisciplinary team should carefully counsel patients the pros and cons of omitting radiation therapy in this setting and balance patients’ risks of locoregional recurrence and other risk factors of developing BCRAL.

The expert panel recommended that patients who receive postoperative radiotherapy should be prioritized for prevention strategies for BCRAL. While there is an well-established relationship between radiotherapy and the development of BCRAL based on many cohort studies as well as the landmark RCTs of MA20 and the EORTC 22922,^15^^,^^45^^,^^46^ there is a growing body of evidence that the risk of BCRAL after radiotherapy depends on the type of axillary surgery that was performed. In a network meta-analysis, the additional risk of locoregional radiation on the development of BCARL was mainly observed in patients who received ALND, but not in those who had SLN biopsy or axillary lymph node sampling.^47^ In a prospective study of 1815 patients, Naoum et al. further showed that the type of axillary surgery is the main driver of the development of BCRAL and not postoperative radiation therapy.^48^ Regardless of whether patients had ALND or SLN biopsy, the study showed that there were no additional risks of BCRAL in patients who received locoregional radiation therapy compared to those who received axillary surgery alone. Importantly, after controlling for other risk factors of BCRAL development, patients who received ALND alone had a higher incidence of BCRAL compared to those who received SLN biopsy plus radiation therapy.^48^ In institutions with resource constraints, preventative strategies for BCRAL should be first offered to patients who had ALND plus radiation therapy, followed by patients with ALND alone. Patients with SLN biopsy can be considered to have lower risk of BCRAL, even if they received adjuvant radiation therapy.

To date, whether modifying the radiation technique and coverage can reduce the risk of BCRAL is controversial in view of the discordant results reported in the literature. While Gross et al. showed that patients who received an extended supraclavicular field to include the axilla had a higher risk of BCRAL, Chandra et al. demonstrated that the risks of BCRAL were similar in patients with or without an extended supraclavicular field or the addition of a posterior axillary boost.^49^^,^^50^ This is supplemented by the conflicting results in studies that attempted to correlate the dose to subregions of the axilla and the development of BCRAL. Gross et al. reported that the axillary-lateral thoracic vessel juncture (ALTJ) was a structure that highly correlated with the development of BCRAL.^51^ However, a recent validation study by Healy et al. did not show a similar relationship.^52^ Both of these studies included patients who had postoperative radiation therapy after ALND. Future dosimetric correlation should be performed in patients who develop BCRAL after axillary radiation therapy alone to avoid the confounding effect of ALND. Before these results are available, dosimetric constraints to the axillary substructures should not be applied as this may compromise the radiation coverage of the axilla in early breast cancer patients with a positive SLN biopsy who are offered axillary radiation in lieu of ALND.

Based on the systematic review of risk factors by Shen et al., our expert panel also recommended that patients who had a greater number of axillary lymph nodes removed should be prioritised in receiving interventions to prevent BCARL. With advances in surgical techniques, there is a suggestion that it is how the lymph nodes are removed that contribute to the development of BCRAL rather than the total number of lymph nodes. In a prospective lymphoedema screening trial involving 2623 patients, Naoum et al. reported that the risk of BCRAL did not increase by the number of lymph nodes removed in both the SLN biopsy (N = 1914) and ALND (N = 709) groups.^53^ In the 690 patients who had 3 to 11 lymph nodes removed by either type of axillary surgery, ALND remained significantly associated with the development of BCRAL (HR: 4.2, p < 0.0001) after controlling for other risk factors of BCRAL such as BMI, radiotherapy, age and type of breast surgery.^53^ Importantly, this analysis showed that patients with clinical N2 disease who had more lymph nodes removed did not have better locoregional control and overall survival. Results of ongoing RCTs (NSABP B-51, MA19, EUBREAST-01) on de-escalation of axillary surgery are eagerly awaited to evaluate the oncological safety of these treatment approaches.

Prophylactic lymphatic reconstruction and axillary reverse mapping are sophisticated surgical techniques that require specialized training to perform. Concerns that axillary reverse mapping may compromise oncologic safety may also be a reason that limit its worldwide adoption.^54^ The systematic reviews by Cook et al. and Co et al. provide the highest level of evidence regarding the safety and efficacy of these techniques to prevent BCRAL.^11^^,^^12^ In the first round of the survey, statements that recommended offering prophylactic lymphatic reconstruction and axillary reverse mapping did not reach consensus. Some experts reflected that these statements were too strong that the data were not sufficient to recommend these treatments as a standard of care. When statements were amended to recommend these surgical techniques as “options,” consensus was achieved. Preliminary results of an ongoing RCT further confirmed the efficacy of prophylactic lymphatic reconstruction.^55^ New surgical approaches of the procedure, such as use of a lower extremity vein graft, was shown to reduce the intraoperative abort rate from 14% to 0%.^56^ These evidence may promote prophylactic lymphatic reconstruction to be adopted in more centres worldwide. Nevertheless, more studies are needed to determine the cost-effectiveness of these surgical approaches, especially when evidence-based methods that are less complicated and resource intensive such as surveillance programs and prophylactic compression sleeves are available. Furthermore, strategies to reduce the impact of nodal irradiation on patients who received lymphatic reconstruction have to be studied, as the incidence of BCRAL increased from 2.1% in patients who did not receive radiotherapy to 10.3% in those who received radiotherapy.^57^ A dosimetric study by Spiegel et al. showed that the immediate lymphatic reconstruction anastomoses were often irradiated with substantial doses even if they were not targeted intentionally.^58^ Long term follow-up data correlating the dose to this structure and the incidence of BCRAL will inform whether using advanced techniques to spare this structure from radiation will be beneficial.

A strength of our Delphi study is that the statements were all generated based on studies with high level evidence. This increases the reliability of the recommendations of the expert panel. Moreover, we incorporated resource considerations in our statements. We specifically invited experts to rank the risk factors that they believed were the most important to guide how patients should be prioritised for preventative strategies. This enhances the applicability across different countries and regions, regardless of their resource settings. Furthermore, our panel comprised of experts representing high-income and middle-to low-income countries, practitioners working in academic and community settings, MASCC and non-MASCC members and a wide range of disciplines involved in the care of breast cancer or BCRAL. Importantly, we also actively engaged with a patient advocate to take on a lead role in our steering committee. She participated in all aspects of the study, including the design of the survey, reviewed and approved all statements at every round of our survey.

However, our study has several limitations. First, as we employed snowball sampling in our Delphi study, some participants were invited based on referrals from other experts. This could have resulted in over-representation of experts from a particular discipline or country/region. Also, patients who work together may share the similar opinion or clinical experience and therefore responses could be biased. To overcome this shortcoming, we attempted to broaden representation by reaching out to the MASCC Oncodermatology and Survivorship study group members, as well as international lymphoedema societies. Second, as our Delphi study included a range of interventions for the prevention of BCRAL across different disciplines, some experts may not be as familiar in certain aspects of care compared to others. For example, physiotherapists may not be familiar with the technical aspects of axillary radiotherapy, while oncologists may not have experience in using BIS. Nevertheless, as the six original articles that the Delphi study was based on were presented to the panel while they filled in the survey, they could rate the statements based on their own critical appraisal of the studies. Third, we added an option for experts to indicate whether they “do not know or not within scope of practice to judge” to better understand the position of the participants in the second round of the survey. We recognise that the participants' responses may be different if they were given this choice in the first round and this could have affected the consensus statements. Nevertheless, there were still at least 30 experts who rated each of the statements, which is considered an optimum number for Delphi studies.^59^ This method was helpful as it allowed raters to decide whether they had the expertise to rate the statement or not. Fourth, a systematic review of the literature was not performed prior to the Delphi study, which could result in bias in the articles that were chosen as the basis of the statements. The steering committee decided that there was an urgent need to develop a clinical practical guidance given the strength of the evidence of the RCTs and systematic reviews identified in our search. We recognise that our approach may not encompass all the emerging evidence for the prevention of BCRAL reported in the literature. Lastly, inherent to all modified Delphi studies, the active role of the steering group to modify statements based on participants’ feedback to achieve consensus may introduce bias. To have a balanced view, the MASCC Oncodermatology and Survivorship study groups convened an international steering group with a wide range of expertise including oncologists, nurses, researchers and a patient advocate that reviewed the questionnaire and statements before proceeding to the next round.

Moving forward, it is important to assess patients' preferences for these interventions. Prospective surveillance programs may add financial costs and time burden to patients, especially for patients who live far away from their healthcare facilities. Koelmeyer et al. designed a home-based prospective surveillance program utilizing BIS, which was shown to be well-accepted by patients and feasible.^60^ The results of an on-going RCT evaluating the efficacy of home-based prospective surveillance using self-measured arm circumference and BIS are eagerly awaited (NCT04522648).^61^ Further investigations comparing the efficacy of these programs to hospital-based models are also warranted.

Prophylactic compression sleeves likewise may not be accepted by some patients. A cross-sectional survey by Blom et al. demonstrated that some patients with mild BCRAL experienced practical and emotional issues related to the sleeve, although overall HRQoL scores were not significantly different compared to patients not wearing the sleeves.^62^ A patient preference survey should be performed amongst newly diagnosed patients to understand their perspectives. Institutions that are already implementing these measures should regularly collect patient feedback to improve care and consider alternative interventions if patients are not complying with them.

It is important to highlight that interventions recommended in this Delphi consensus and that of Martinez-Jaimez et al. do not guarantee complete prevention of BCRAL, but only reduce the risk of its development. In the studies that our Delphi consensus was based on, a considerable proportion of patients still develop BCRAL despite these interventions. For example, 14% of patients in the prophylactic compression sleeve group had relative arm volume increase at 1 year compared to 25% in the non-sleeve group in the RCT of Paramanadam et al. Raising awareness of BCRAL amongst patients, healthcare professionals and administrators is crucial in ensuring patients with BCRAL receive timely referrals to appropriate care. Basic and translational research to understand the complex pathophysiology of BCRAL are also important to inspire innovative management strategies to be tested in future clinical trials.

The epidemiology of BCRAL will continue to evolve in the next decade with the increased use of highly effective neoadjuvant and adjuvant systemic treatments, partial breast irradiation and (ultra-) hypofractionation radiation schedules. The international community needs to regularly monitor for updates on methods in preventing BCRAL and review whether the interventions recommended in this Delphi study remain effective. Implementing prospective surveillance programs and early intervention was estimated to require less costs compared to the traditional model of treating patients with late stage BCRAL.^63^ However, if there is an overall declining incidence of BCRAL with advances in anticancer treatments, the number needed to treat may significantly increase and hence render such programs less cost effective. An update of this Delphi study is proposed in 5–10 years to evaluate whether these interventions are still relevant, and whether there are new interventions to be recommended.

In conclusion, this international Delphi study generated practical recommendations for evidence-based practices to prevent BCRAL. An individualised approach taking into account patients' preferences, risk factors for BCRAL, availability of different treatment options and expertise of the healthcare team is paramount to ensure all patients at risk will be offered preventive interventions for BCRAL, regardless of where they are receiving care, while the contemporary vast majority of patients who are not at risk for BCRAL are spared the psychological, physical and financial burden.

## Contributors

HCYW, MPW, AWC, ND, PB, MB, JRW, CvdH, MF, EC and RJC were responsible for the conceptualisation, data curation, formal analysis, investigation, methodology and project administration. EC and RJC supervised the project. HCYW wrote the original draft. HCYW, MPW, AWC, ND, PB, MB, JRW, CvdH, MF, EC, RJC, MA, BAA, SB, KB, PB, YB, DC, SMC, YC, NSJC, JIC, YPC, KC, ED, PH, SH, KH, SFL, MH, PJ, YK, DK, HK, CK, JL, MYL, ML, BL, PM, SM, IM, GNM, MMcN, TM, LELM, MOg, MOs, SP, PP, BSR, AR, AR, AR-G, JR, NS, CBSII, MS, KT, LFNT, MTe, MTr, KHW, and KY critically revised the manuscript for intellectual content. HCWY, MPW, AWC, ND, EC, RJC had full access to the data of the study. All authors read and approved the final version of the manuscript.

## Data sharing statement

The dataset generated from this study are available from the corresponding author on reasonable request.

## Declaration of interests

Elizabeth Dylke received support to attend Australasian Lymphology Association Symposium 2022 and 2023, and is the President and Director of Australasian Lymphology Association and Secretary of the Council of the Deans of Physiotherapy Australia and New Zealand; Yuichiro Kikawa received payment or honoraria for lectures, presentations, speaker bureaus, manuscript writing or education events from Eisai, Lilly, Chugai, Pfizer, Daiichi Sankyo and Novartis; Michael Lock received consulting fees from Bayer and Tersera, payment or honoraria for lectures, presentations, speaker bureaus, manuscript writing or education events from Knight Therapeutics, Abbvie and Eisai, and have stock options from Myovant; Icro Meattini received honoraria for lectures from Eli Lily, AstraZeneca, SeaGen, Gilead, Daiichi, Sankyo, Pfizer and Novartis; Tammy Mondry received consulting fees from Teladoc Health, and payment for honoraria from Klose Training; Abram Recht received grants or contracts from the Joint Centre for Radiation Therapy Foundation paid to his institution, received consulting fees from eviCore healthcare and EXACT Sciences Corporation, and has stock ownership of Imagine Scientific, Inc.; Jolien Robijns received grants or contracts from Kom op Tegen Kanker and Limburgs Kankerfonds paid to Hasselt University. All other authors declare no competing interests.

## Appendix A Supplementary data

Appendix 1

Expert Panel Names
