# Supplementary material for: Multinational Association of Supportive Care in Cancer (MASCC) clinical practice guidance for the prevention of breast cancer-related arm lymphoedema (BCRAL): international Delphi consensus-based recommendations
Source: eClinicalMedicine. 2024 Feb 2;68:102441. doi: 10.1016/j.eclinm.2024.102441 (PMC10850412; doi:10.1016/j.eclinm.2024.102441)
Supplement: Supplementary information A [file mmc1.docx]

**Supplementary Evidence Sheet**

***Part 1: Risk factors for breast cancer related arm lymphedema***

^a^Pooled odds ratio of risk factors for breast cancer related arm lymphedema in systematic review by Shen et al.

| **Risk factor** | **Pooled Odds ratio in systematic review (95% CI)** |
| --- | --- |
| **Treatment related** | |
| Mastectomy (vs. breast conservation therapy) | 1.30 (1.06-1.58) |
| Axillary lymph node dissection (vs. sentinel lymph node biopsy) | 3.10 (2.48-3.87) |
| Use of chemotherapy (vs. No chemotherapy) | 1.36 (1.15-1.60) |
| Use of post-operative radiotherapy (vs. No radiotherapy) | 1.99 (1.65-2.39) |
| Number of lymph nodes dissected (continuous) | 1.04 (1.03-1.06) |
| Postoperative complications (vs. No complications) | 2.04 (1.51-2.76) |
| Relative arm volume increase >5% 1 month post op (vs < 3%) | 5.54 (3.85-7.9) |
| **Patient related** | |
| Body mass index (continuous) | 1.06 (1.04-1.09) |
| Black race (vs. White) | 1.26 (1.02-1.56) |
| Hypertension (vs. No hypertension) | 2.19 (1.37-3.51) |
| **Disease related** | |
| TNM Stage III (vs. Stage I-II) | 1.34 (1.34-2.61) |
| Tumour size (continuous) | 3.17 (1.70-5.92) |

^b^ Pooled odds ratio of body mass index as risk factor for breast cancer related arm lymphedema in systematic review by Shen et al.

| **BMI** | **Pooled Odds ratio in systematic review (95% CI)** |
| --- | --- |
| Continuous (per 1 kg /m^2^) | 1.06 (1.04-1.09) |
| ≥ 25 kg /m^2^ (vs <25 kg /m^2^) | 2.09 (1.45-3.02) |
| ≥ 30 kg/ m^2^ (vs <30 kg/m^2^) | 1.93 (1.62-2.31) |
| 25-29.9 kg/m^2^ (vs <25 kg/m^2^) | 1.30 (1.14-1.49) |

^c^Pooled odds ratio of timing of chemotherapy as risk factor for breast cancer related arm lymphedema in systematic review by Shen et al.

| **Timing of chemotherapy** | **Pooled Odds ratio in systematic review (95% CI)** |
| --- | --- |
| Use of chemotherapy | 1.36 (1.15-1.60) (vs. No chemotherapy) |
| Neoadjuvant chemotherapy | 2.21 (1.50-3.26) (vs No neoadjuvant chemotherapy) |
| Adjuvant chemotherapy | 1.15 (0.800, 1.66) (vs No adjuvant chemotherapy) |

^d^Pooled odds ratio of type of chemotherapy as risk factor for breast cancer related arm lymphedema in systematic review by Shen et al.

| **Type of chemotherapy** | **Pooled Odds ratio in systematic review (95% CI)** |
| --- | --- |
| Chemotherapy including taxane (vs chemotherapy without taxane) | 2.21(1.99-2.47) |
| Taxane based chemotherapy (vs no chemotherapy) | 1.70 (1.30-2.21) |
| Non-taxane chemotherapy (vs no chemotherapy) | 0.818 (0.41-1.64) |

^e^Pooled odds ratio of number of lymph nodes as risk factor for breast cancer related arm lymphedema in systematic review by Shen et al.

| **Number of lymph node dissected** | **Pooled Odds ratio in systematic review (95% CI)** |
| --- | --- |
| Continuous | 1.04 (1.03-1.06) |
| ≥ 10 (vs <10) | 1.44 (1.01-2.05) |
| ≥15 (vs <15) | 1.13 (1.03-1.25) |

^f^Pooled odds ratio of type of radiotherapy coverage as risk factor for breast cancer related arm lymphedema in systematic review by Shen et al.

| Radiotherapy coverage | **Pooled Odds ratio in systematic review (95% CI)** |
| --- | --- |
| Axillary radiotherapy (vs No radiotherapy) | 2.13 (1.54-2.96) |
| Breast / chest wall radiotherapy (vs No radiotherapy) | 1.43 (0.75-2.77) |
| Breast / chest wall + supraclavicular radiotherapy (vs No radiotherapy) | 1.288 (0.43-3.83) |
| Breast / chest wall + supraclavicular +axillary radiotherapy ( vs No radiotherapy) | 1.30 (0.62-2.72) |

***Part 2: Prospective surveillance***

^a^In the systematic review by Rafn et al, participation in prospective surveillance with early management reduced the risk of chronic breast cancer related arm lymphedema versus usual care (relative risk 0.31; 95% CI, 0.10 to 0.95; two randomized controlled trials; N = 106).

^b^Measurement methods of surveillance programs in included studies in systematic review by Rahn et al.

| **Measurement method in surveillance program** | **Number of studies in the systematic review by Rahn et al.** |
| --- | --- |
| Bioimpedance spectroscopy | 10 |
| Circumference measurement | 4 |
| Perometry | 4 |
| Water displacement | 4 |
| Others | 6 |

^c^Different criteria are used to determine when patients should be initiated treatment and how chronic lymphedema is diagnosed in the studies included in Rafn et al.


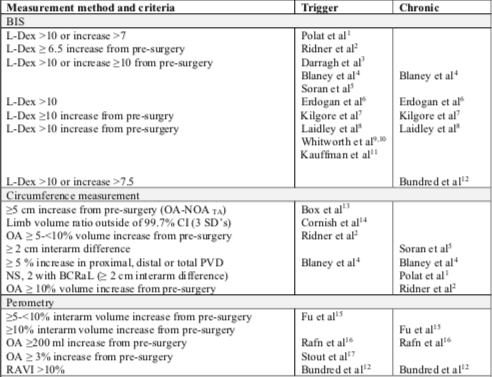


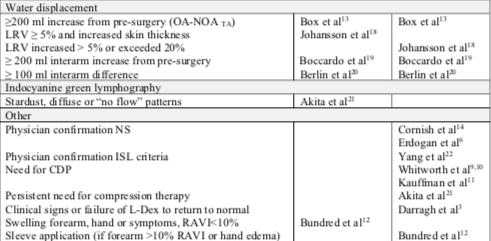

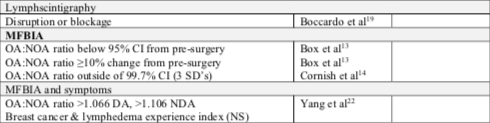


^d^In the systematic review by Rafn et al, 95% of studies had the first post-operative assessment within 3 months after surgery.

^e^In the systematic review by Rafn et al, prospective surveillance programs included presurgical measurements of breast cancer related arm lymphedema in all studies except for two.

^f^In the systematic review by Rafn et al., 18 studies (86%) performed at least three follow-up measures at regular intervals during the first year after surgery. Twelve studies (57%) evaluated prospective surveillance and early management programs lasting more than 1 year, of which 10 studies performed annual or biannual measures for the duration of the program.

^g^In the systematic review by Rafn et al, 11 studies (52%) describe a prospective surveillance program with last follow-up ≥ 24 months post-surgery.

^h^All studies in the systematic review by Rahn et al. involved surveillance protocol performed solely by healthcare professionals.

^i^In the systematic review by Rahn et al., when early lymphedema was detected in surveillance programs, compression garments were applied in 17 (81%) studies.

^j^Six out of 17 studies specified the duration of compression sleeve applied when lymphedema is detected in a surveillance program. Compression sleeves were applied for 4 weeks in 5 studies and 4 to 6 weeks in 1 study

***Part 3: Prophylactic use of compression sleeves***

^a^In the study by Paramanandam et al., prophylactic use of compression sleeves resulted in lower risk in arm swelling compared to the control group at 1 year (HR 0.61; 95% CI 0.43-0.85, P=0.004 using bioimpedance spectroscopy; HR 0.56 95% CI 0.33-0.96; P = 0.034 using relative arm volume measurements).

^b^In the study by Paramanandam et al., prophylactic compression sleeves were applied from first post-operative day until 3 months after the completion of adjuvant treatments (excluding hormonal treatments).

^c^In the study by Paramanandam et al., prophylactic arm sleeves were applied at least 8 hours.

^d^In the study by Paramanandam et al., the pressure of the prophylactic arm sleeves was 20-25 mmHg.

^e^In the study by Paramanandam et al., patients in the prophylactic arm sleeves group were assessed every 6 months for any lymphedema

^f^In the study by Paramanandam et al., patients were assessed for breast cancer related arm lymphedema using bioimpedance testing and relative volume measurements compared to the non-operative arm during and after application of prophylactic compression sleeves

^g^In the study by Paramanandam et al., diagnosis of first breast cancer related arm lymphedema event (arm swelling) is by bioimpedance testing or increase in relative arm volume by ≥10%

***Part 4: Axillary radiation instead of axillary lymph node dissection for clinical node negative patients with positive sentinel lymph node biopsy***

^a^In the AMAROS study, axillary lymph node dissection was associated with a higher risk of lymphedema compared to axillary radiation for patients with cT1-2, node-negative breast cancers with a positive sentinel lymph node biopsy (24.5% *v* 11.9%; *P* < .001). The axillary recurrence risks were similar.

^b^The AMAROS study included patients who had mastectomy or breast conservation therapy. More than 80 % of patients received breast conservation therapy.

^c^The AMAROS study included patients who had any number of positive sentinel lymph nodes. Around 1% of patients had four or more positive lymph nodes on sentinel lymph node biopsy (pN2).

^d^The AMAROS study included patients with tumours of any grade. Around 30% of patients had grade 3 disease.

^e^The subgroup distributions of patients according to hormonal receptor and HER2 status were not reported in the AMAROS study.

^f^The AMAROS study included patients with any number of sentinel lymph nodes removed. Around 40% of patients only had 1 sentinel lymph node removed.

^g^The AMAROS study allowed irradiation of the internal mammary chain when axillary radiation is given. Around 10% of patients who received axillary radiation were given radiation to the internal mammary chain.

^h^The AMAROS study included patients with positive sentinel lymph nodes of any size. Around 40% of patients who received axillary radiation had micrometastases or isolated tumour cells.

^i^Second primary cancers were observed more in the axillary radiation arm compared to the axillary lymph node dissection arm in the AMAROS study.

^j^Patients received 50 Gy in 25 daily fractions over 5 weeks in the axillary radiation arm of the AMAROS study.

***Part 5: Prophylactic lymphatic reconstruction***

^a^The systematic review by Cook et al. showed that immediate lymphatic reconstruction may decrease risk of development of lymphedema to 6.6%.

^b^In the pooled analysis of patients in the systematic review by Cook et al., the mean body mass index (BMI) was 27.0kg/m^2^, a mean of 16.0 lymph nodes were removed at the time of axillary lymph node dissection and more than 70% underwent post-mastectomy radiation therapy.
