# Supplementary material for: Multinational Association of Supportive Care in Cancer (MASCC) clinical practice guidance for the prevention of breast cancer-related arm lymphoedema (BCRAL): international Delphi consensus-based recommendations
Source: eClinicalMedicine. 2024 Feb 2;68:102441. doi: 10.1016/j.eclinm.2024.102441 (PMC10850412; doi:10.1016/j.eclinm.2024.102441)
Supplement: Supplementary information B [file mmc2.pdf]

BCRaL Information Sheet

PARTICIPANT INFORMATION SHEET AND CONSENT FORM

TITLE: Multinational Association of Supportive Care in Cancer (MASCC) Led International Delphi Consensus on Measures to Prevent Breast Cancer-Related Arm Lymphoedema - Joint Project from Oncodermatology and Survivorship Study Groups

Principal Investigators	Affiliations
**Dr Matthew Wallen**<br>Email: matthew.wallen@flinders.edu.au	College of Nursing and Health Sciences, Flinders University
**Miss Yani Dick**<br>Email: yani.dick@flinders.edu.au	College of Nursing and Health Sciences, Flinders University
**Professor Raymond Chan**<br>Email: raymond.chan@flinders.edu.au	College of Nursing and Health Sciences, Flinders University
**Dr Henry Wong**<br>Email: henrywong3011@gmail.com	Department of Oncology, Princess Margaret Hospital, Hong Kong
**Dr Adrian Chan**<br>Email: ac_wai@hotmail.com	Department of Clinical Oncology, Tuen Mun Hospital, Hong Kong
**Professor Edward Chow**<br>Email: edward.chow@sunnybrook.ca	Sunnybrook Health Sciences Centre, University of Toronto
**Associate Investigators**	
**Monique Bareham**<br>Email: lymphadvocate@gmail.com	Consumer Advocate (South Australia), Lymphoedema Association South Australia
**Professor Margaret Fitch**<br>Email: marg.i.fitch@gmail.com	University of Toronto
**Dr Corina van den Hurk**<br>Email: c.vandenhurk@iknl.nl	Netherlands Comprehensive Cancer Organisation
**Dr Julie Ryan Wolf**<br>Email:<br>Julie_Ryan@URMC.Rochester.edu	University of Rochester

Description of the study

Breast cancer-related arm lymphoedema (BCRaL) is a common, long-term complication of anti-cancer treatments, which occurs in approximately one in five breast cancer survivors. As chronic lymphoedema is difficult to treat and significantly affects patients' quality of life, prophylactic management to prevent lymphoedema, early detection of subclinical lymphoedema and halting its progression are important.

Axillary lymph node dissection is an important treatment-related risk factor of lymphoedema, with a significant association identified with the number of lymph nodes removed. Recent evidence shows that clinically node-negative patients who were found to have positive lymph nodes at sentinel lymph node biopsy should be offered axillary radiation instead of axillary lymph node dissection. Moreover, for patients who are clinically node-positive or have a positive sentinel lymph node biopsy, immediate lymphatic reconstruction performed in the same session as axillary lymph node dissection or axillary reverse mapping are promising surgical techniques in reducing the risks of lymphoedema.

Measures taken after surgery are also effective in lowering the risks of chronic lymphoedema. A recent review showed that prospective surveillance followed by early intervention could effectively reduce the incidence of chronic lymphoedema. Additionally, a recent randomised control trial showed that the use of prophylactic compression sleeves before symptoms develop can decrease arm swelling and reduce the frequency of chronic lymphoedema.

Healthcare professionals managing breast cancer and lymphoedema may be uncertain about how to put implement recommendations into clinical practice. Given the strength of recent evidence and resource limitations in the health system, which patients should be given these treatments and how they should be implemented remain unclear.

Purpose of the current study

We aim to use a Delphi consensus methodology to:

1. seek consensus on whether practices to prevent lymphoedema are recommended,
2. determine which patients and how to implement these treatments if they are recommended, and
3. understand how further research should be conducted to provide more guidance if these treatments are not recommended.

Benefits of the study

There will be no direct benefit to you taking part in this study but the sharing of your views and experiences will help us establish consensus on measures to prevent breast cancer-related arm lymphoedema, and to inform future research in the area.

Participant involvement and potential risks

If you agree to participate in the research study, you will be asked to complete two or three online surveys that ask for your views about a series of recommended statements developed by the investigating team based on recently published evidence:

**Study Overview**	**Reference**
Systematic review on the risk factors of BCRaL	Shen A, Lu Q, Fu X, et al. Risk factors of unilateral breast cancer-related lymphedema: an updated systematic review and meta-analysis of 84 cohort studies. *Support Care Cancer*. 2023;31(1):18. doi:10.1007/s00520-022-07508-2
A systematic review and meta-analysis on prospective surveillance for BCRaL	Rafn BS, Christensen J, Larsen A, Bloomquist K. Prospective Surveillance for Breast Cancer–Related Arm Lymphedema: A Systematic Review and Meta-Analysis. *JCO*. 2022;40(9):1009-1026. doi:10.1200/JCO.21.01681
A randomised controlled trial on prophylactic compression arm sleeves	Paramanandam VS, Dylke E, Clark GM, et al. Prophylactic Use of Compression Sleeves Reduces the Incidence of Arm Swelling in Women at High Risk of Breast Cancer–Related Lymphedema: A Randomized Controlled Trial. *JCO*. 2022;40(18):2004-2012. doi:10.1200/JCO.21.02567
The ten-year follow-up data of the AMAROS randomised controlled trial	Bartels SAL, Donker M, Poncet C, et al. Radiotherapy or Surgery of the Axilla After a Positive Sentinel Node in Breast Cancer: 10-Year Results of the Randomized Controlled EORTC 10981-22023 AMAROS Trial. *JCO*. 2022:JCO.22.01565. doi:10.1200/JCO.22.01565
A systematic review on prophylactic immediate lymphatic reconstruction to prevent BCRaL	Cook JA, Sinha M, Lester M, Fisher CS, Sen CK, Hassanein AH. Immediate Lymphatic Reconstruction to Prevent Breast Cancer-Related Lymphedema: A Systematic Review. *Advances in Wound Care*. 2022;11(7):382-391. doi:10.1089/wound.2021.0056
Systematic review and pooled analysis on axillary reverse mapping to prevent BCRaL	Co M, Lam L, Suen D, Kwong A. Axillary Reverse Mapping in the Prevention of Lymphoedema: A Systematic Review and Pooled Analysis. *Clinical Breast Cancer*. 2023;23(1):e14-e19. doi:10.1016/j.clbc.2022.10.008

Before participating in the first online survey, you will be provided with the published evidence specified above. Each survey will take around 20 minutes to complete.

The investigators do not foresee any risks or discomfort to participants or researchers who are involved in this research. Inconvenience is the only consequence that could be associated with this research.

However, if you experience feelings of distress because of participation in this study, please let the investigator team know immediately, and they will refer you to receive support.

Withdrawal Rights

You will be required to provide informed consent before participation and will be notified about the commitments you will be expected to fulfil should you agree to participate (the consent form is attached at the end of this information sheet).

Participation in this study is voluntary and you can withdraw at any time during the study. If you take part and later change your mind, you may withdraw at any time without providing an explanation. If you choose to withdraw, you will not be penalised or made to feel uncomfortable.

To withdraw, please contact the Dr Matthew Wallen using the details provided above. Alternatively, you may just refuse to answer any questions at any time (or close the internet browser or leave the online survey). Any data collected up to the point of withdrawal will be securely destroyed.

Confidentiality and Privacy

Only investigators listed on this participant information sheet have access to the individual information provided by you. Privacy and confidentiality will be assured at all times.

The findings from the initial round of surveys (i.e., the list of statements that reached consensus in round 1) will be circulated in an additional round of surveys. However, this this will not include any identifiable data.

The research outcomes may be presented at conferences, written up for publication or used for other research purposes as described in this participant information sheet. However, the privacy and confidentiality of individuals will be protected at all times. You will not be named, and your individual information will not be identifiable in any research products without your explicit consent.

No data will be shared or used in future research projects.

Data storage

The information collected may be stored securely on a password protected computer and/or Flinders University server throughout the study. At the completion of the study, participants will be allocated a unique study ID which will be generated and securely stored separately before all participant identifiers are purged, rendering the data re-identifiable. All data will be securely transferred to and stored at Flinders University for five years after publication of the results. Following the required data storage period, all data will be securely destroyed according to university protocols.

Investigators will ensure that data is collected and stored in accordance with relevant international jurisdictions where required (e.g., the EU General Data Protection Regulation).

How will I receive feedback?

On project completion, a short summary of the outcomes will be provided to all participants via email or published on Flinders University's website (which participants will be notified of).

Ethics Committee Approval

The project has been approved by Flinders University's Human Research Ethics Committee (project number 5937).

Queries and Concerns

Queries or concerns regarding the research can be directed to the research team. If you have any complaints or reservations about the ethical conduct of this study, you may contact the Flinders University's Research Ethics & Compliance Office team via telephone (08) 8201 2543 or email human.researchethics@flinders.edu.au.

Thank you for taking the time to read this information sheet. You can download a copy of the information sheet here. If you accept our invitation to be involved, please complete the Consent Form on the next page.

BCRaL - Consent

Flinders University

Caring Futures Institute

PARTICIPANT INFORMATION SHEET AND CONSENT FORM

Consent Statement

1. I have read the information provided and I understand I am being asked to provide informed consent to participate in this research study. I understand that I can contact the research team if I have further questions about this research study.

2. I am not aware of any condition that would prevent my participation, and I agree to participate in this project.

3. I understand that I am free to withdraw at any time during the study.

4. I understand that I can contact Flinders University's Research Ethics & Compliance Office if I have any complaints or reservations about the ethical conduct of this study.

5. I understand that my involvement is confidential, and that the information collected may be published. I understand that I will not be identified in any research products.

If you wish to participate, please select 'I agree' below. By selecting 'I agree', you confirm that you have read and understood the information provided and consent to participating in the study.

- ◯ I AGREE
- ◯ I DO NOT AGREE

Please provide your contact details below before proceeding to the survey. These will only be used to facilitate the distribution of subsequent surveys.

First Name

[ ]

Surname

[ ]

Email Address

[ ]

BCRaL - Introduction / Instructions

Thank-you for consenting to participate in the study titled Multinational Association of Supportive Care in Cancer (MASCC) Led International Delphi Consensus on Measures to Prevent Breast Cancer-Related Arm Lymphoedema - Joint Project from Oncodermatology and Survivorship Study Groups.

On behalf of the study groups, I would like to formally thank-you for time, expertise, and contribution.

Prior to commencing the survey, we strongly encourage that you read through the following six (6) studies which were used in the development of the statements:

Shen et al. 2023
Rafn et al. 2022
Paramanandam et al. 2022
Bartels et al. 2022
Cook et al. 2022
Co et al. 2023

In addition to the above, we have also provided the following resources to facilitate answering the questions within the survey:

- A copy of the statements within the first round survey
- Supplementary information from each specific study

Please ensure that you are familiar with these resources before moving to the survey. You are welcome to go back to these resources during the survey.

Once you are happy to proceed, please commence the survey by clicking on the bottom-right arrow at the bottom of the screen.

BCRaL - Participant Demographics

What is your age, in years?

- ◯ 18-24
- ◯ 25-34
- ◯ 35-44
- ◯ 45-54
- ◯ 55-64
- ◯ 65-74
- ◯ 75-84
- ◯ 85 and over

What is your gender identity?

- ◯ Male
- ◯ Female
- ◯ Non-binary / third gender
- ◯ Prefer to self-describe

- ◯ Prefer not to say

What country do you currently work in?

What is your primary occupation? (Please select all that apply)

- ☐ Medical Oncologist
- ☐ Radiation Oncologist
- ☐ Surgeon
- ☐ General Practitioner / Physician
- ☐ Medical Doctor (please specify)
- ☐ Registered Nurse (general)
- ☐ Registered Nurse (specialist) (please specify)
- ☐ Physiotherapist / Physical Therapist
- ☐ Occupational Therapist
- ☐ Researcher / Academic
- ☐ Other (please specify)

How many years experience do you have working in breast cancer / lymphoedema care?

- ◯ < 1 year
- ◯ 1-5 years
- ◯ 5-10 years
- ◯ 10-15 years
- ◯ 15-20 years
- ◯ 20-25 years
- ◯ 25-30 years
- ◯ 30-35 years
- ◯ 40-45 years
- ◯ 45-50 years
- ◯ > 50 years

Part 1 - Closed Questions - Risk for breast cancer-related arm lymphoedema

Part 1 - Risk factors for breast cancer-related arm lymphoedema

The following questions / statements are based on the systematic review and meta-analysis ***Risk factors of unilateral breast cancer-related lymphedema: an updated systematic review and meta-analysis of 84 cohorts studies*** by Shen et al. 2023.

Please see supplementary sheet for details of evidence (letters in parentheses denotes the evidence that the question / statement is based on).

Please sort the following risk factors according to their importance in selecting patients for prophylactic management of lymphoedema (a).

	High Importance	Medium Importance	Low Importance
Mastectomy	○	○	○
Axillary lymph node dissection	○	○	○
Use of chemotherapy	○	○	○
Use of post-operative radiotherapy	○	○	○
Greater number of lymph nodes dissected	○	○	○
Presence of post-operative complications	○	○	○
Relative arm volume increase 1 month after surgery	○	○	○
High body mass index	○	○	○
Black race	○	○	○
Presence of hypertension	○	○	○
Higher tumour (TNM) stage	○	○	○
Greater tumour size	○	○	○

Please drag and drop the five (5) most important risk factors in selecting patients for prophylactic management of lymphoedema and rank them according to the level of importance (a)

Items

Mastectomy

Axillary lymph node dissection

Use of chemotherapy

Use of post-operative radiotherapy

Number of lymph nodes dissected

Post-operative complications

Relative arm volume increase

Body mass index (BMI)

Race

Hypertension

Tumour stage

Priority risk factor 1 (most important)	Priority risk factor 2	Priority risk factor 3	Priority risk factor 4	Priority risk factor 5

Tumour size

Please indicate your level of agreement or disagreement to the following statements:

	Completely disagree	Disagree	Neutral	Agree	Completely agree
When there are resource constraints, patients who present with a higher BMI (BMI ≥ 30 kg / m2) should be prioritised over patients with BMI < 30 kg / m2 when selecting patients for prophylactic management of lymphoedema (b)	○	○	○	○	○
When there are resource constraints, patients who received neoadjuvant chemotherapy should be prioritised over patients who receive adjuvant chemotherapy when selecting patients for prophylactic management of lymphoedema (c)	○	○	○	○	○
When there are resource constraints, patients who receive taxane based chemotherapy should be prioritised over patients who receive other types of chemotherapy when selecting patients for prophylactic management of lymphoedema (d)	○	○	○	○	○
When there are resource constraints, patients who had ≥ 15 axillary lymph nodes removed in axillary dissection should be prioritised over patients who had less lymph nodes removed when selecting patients for prophylactic management of lymphoedema (e)	○	○	○	○	○
When there are resource constraints, patients who received axillary radiation should be prioritised over patients who receive radiation to the breast / chest wall +/- the supraclavicular fossa when selecting patients for prophylactic management of lymphoedema (f)	○	○	○	○	○

Part 1 - Open Responses - Risk factors for breast cancer-related arm lymphoedema

Please state your reasons that you 'disagree' or 'strongly disagree' with the statement ***"When there are resource constraints, patients who present with a higher BMI (BMI ≥ 30 kg / m2) should be prioritised over patients with BMI < 30 kg / m2 when selecting patients for prophylactic management of lymphoedema"***

Please state your reasons that you 'disagree' or 'strongly disagree' with the statement ***"When there are resource constraints, patients who received neoadjuvant chemotherapy should be prioritised over patients who receive adjuvant chemotherapy when selecting patients for prophylactic management of lymphoedema"***

Please state your reasons that you 'disagree' or 'strongly disagree' with the statement ***"When there are resource constraints, patients who receive taxane based chemotherapy should be prioritised over patients who receive other types of chemotherapy when selecting patients for prophylactic management of lymphoedema"***

Please state your reasons that you 'disagree' or 'strongly disagree' with the statement ***"When there are resource constraints, patients who had ≥ 15 axillary lymph nodes removed in axillary dissection should be prioritised over patients who had less lymph nodes removed when selecting patients for prophylactic management of lymphoedema"***

Please state your reasons that you 'disagree' or 'strongly disagree' with the statement ***"When there are resource constraints, patients who received axillary radiation should be prioritised over patients who receive radiation to the breast / chest wall +/- the supraclavicular fossa when selecting patients for prophylactic management of lymphoedema"***

Part 2 - Close Questions - Prospective surveillance

Part 2 - Prospective surveillance

The following questions are based on the study ***Prospective Surveillance for Breast Cancer–Related Arm Lymphedema: A Systematic Review and Meta-Analysis*** by Rafn et al. 2022

Please see supplementary sheet for details of evidence (letters in parentheses denotes the evidence that the statement is based on).

Please indicate your level of agreement or disagreement to the following statements:

	Completely disagree	Disagree	Neutral	Agree	Completely agree
A prospective surveillance program is recommended to reduce risks of chronic lymphoedema after breast cancer surgery where feasible and resources allow (a)	○	○	○	○	○
In a prospective surveillance program, bioimpedance spectroscopy should be the preferred method to identify patients with subclinical / early stage lymphoedema for early treatment (b)	○	○	○	○	○
In a prospective surveillance program, arm circumference (or volumetric) or lymphangiography / lymphoscintigraphy measurement is an alternative method to identify patients with subclinical / early stage lymphoedema for early treatment when bioimpedance spectroscopy is not available or there are resource limitations (b)	○	○	○	○	○
In a prospective surveillance program, treatment is triggered when the bioimpedance spectroscopy score is L-Dex > 10 (c)	○	○	○	○	○
In a prospective surveillance program, treatment is triggered when a difference in volume measurements of ≥ 5 but < 10% is seen compared to pre-surgery values (c)	○	○	○	○	○
In a prospective surveillance program, treatment should be triggered by any patient-reported arm symptoms (e.g. swelling, heaviness, tightness, and numbness) (c)	○	○	○	○	○

	Completely disagree	Disagree	Neutral	Agree	Completely agree
In a prospective surveillance program, the diagnosis of chronic lymphedema is made when a difference in bioimpedance spectroscopy scores is L-Dex > 10 compared to pre-surgery values (c)	○	○	○	○	○
In a prospective surveillance program, the diagnosis of chronic lymphoedema is made when a difference in volume measurements is ≥ 10% compared to pre-surgery values (c)	○	○	○	○	○
In a prospective surveillance program, the diagnosis of chronic lymphoedema is made when there are persistent symptoms despite initial treatments (c)	○	○	○	○	○
Prospective surveillance is recommended to start within 3 months after surgery (d)	○	○	○	○	○
Presurgical assessment of lymphoedema is required in a prospective surveillance program for better comparison of measurements after surgery (e)	○	○	○	○	○
The surveillance interval for a prospective surveillance program is recommended to be every 3 to 4 months in the first year then every 6 to 12 months thereafter where feasible and resources allow (f)	○	○	○	○	○
The total duration of surveillance in a prospective surveillance program is receommended to be at least 24 months from surgery where feasible and resources allow (g)	○	○	○	○	○
In a prospective surveillance program, healthcare professionals should conduct the prospective surveillance where feasible and resources allow (h)	○	○	○	○	○
In a prospective surveillance program, patients or family members who receive adequate training should conduct the prospective surveillance where resources are limited (h)	○	○	○	○	○
When subclinical / early stage lymphoedema is detected in a prospective surveillance program, compression garment are recommended for initial treatment (i)	○	○	○	○	○
When subclinical / early stage lymphoedema is detected in a prospective surveillance program, compression sleeves are recommended to be prescribed for 4 to 6 weeks (j)	○	○	○	○	○

Part 2 - Open Responses - Prospective surveillance

Please state your reasons that you 'disagree' or 'strongly disagree' with the statement ***"A prospective surveillance program is recommended to reduce risks of chronic lymphedema after breast cancer surgery where feasible and resources allow"***

Please state your reasons that you 'disagree' or 'strongly disagree' with the statement ***"In a prospective surveillance program, bioimpedance spectroscopy should be the preferred method to identify patients with subclinical / early stage lymphedema for early treatment"***

Please state your reasons that you 'disagree' or 'strongly disagree' with the statement ***"In a prospective surveillance program, arm circumference (or volumetric) or lymphangiography / lymphoscintigraphy measurement is an alternative method to identify patients with subclinical / early stage lymphoedema for early treatment when bioimpedance spectroscopy is not available or there are resource limitations"***

Please state your reasons that you 'disagree' or 'strongly disagree' with the statement ***"In a prospective surveillance program, treatment is triggered when the bioimpedance spectroscopy score is L-Dex > 10"***

Please state your reasons that you 'disagree' or 'strongly disagree' with the statement ***"In a prospective surveillance program, treatment is triggered when a difference in volume measurements of ≥ 5 but < 10% is seen compared to pre-surgery values "***

Please state your reasons that you 'disagree' or 'strongly disagree' with the statement ***"In a prospective surveillance program, treatment should be triggered by any patient-reported arm symptoms (e.g. swelling, heaviness, tightness, and numbness)"***

Please state your reasons that you 'disagree' or 'strongly disagree' with the statement ***"In a prospective surveillance program, the diagnosis of chronic lymphoedema is made when a difference in bioimpedance spectroscopy scores is L-Dex > 10 compared to pre-surgery values"***

Please state your reasons that you 'disagree' or 'strongly disagree' with the statement ***"In a prospective surveillance program, the diagnosis of chronic lymphoedema is made when a difference in volume measurements is ≥ 10% compared to pre-surgery values"***

Please state your reasons that you 'disagree' or 'strongly disagree' with the statement ***"In a prospective surveillance program, the diagnosis of chronic lymphoedema is made when there are persistent symptoms despite initial treatments"***

Please state your reasons that you 'disagree' or 'strongly disagree' with the statement ***"Prospective surveillance is recommended to start within 3 months after surgery"***

Please state your reasons that you 'disagree' or 'strongly disagree' with the statement ***"Pre-surgical assessment of lymphoedema is required in a prospective surveillance program for better comparison of measurements after surgery"***

Please state your reasons that you 'disagree' or 'strongly disagree' with the statement ***"The surveillance interval for a prospective surveillance program is recommended to be every 3 to 4 months in the first year then every 6 to 12 months thereafter where feasible and resources allow"***

Please state your reasons that you 'disagree' or 'strongly disagree' with the statement ***"The total duration of surveillance in a prospective surveillance program is receommended to be at least 24 months from surgery where feasible and resources allow"***

Please state your reasons that you 'disagree' or 'strongly disagree' with the statement ***"In a prospective surveillance program, healthcare professionals should conduct the prospective surveillance where feasible and resources allow"***

Please state your reasons that you 'disagree' or 'strongly disagree' with the statement ***"In a prospective surveillance program, patients or family members who receive adequate training should conduct the prospective surveillance where resources are limited"***

Please state your reasons that you 'disagree' or 'strongly disagree' with the statement ***"When subclinical / early stage lymphoedema is detected in a prospective surveillance program, compression garment are recommended for initial treatment"***

Please state your reasons that you 'disagree' or 'strongly disagree' with the statement ***"When subclinical / early stage lymphoedema is detected in a prospective surveillance program, compression sleeves are recommended to be prescribed for 4 to 6 weeks"***

Part 3 - Closed Questions - Prophylactic use of compression sleeves

Part 3 - Prophylactic use of compression sleeves

Based on the study ***Prophylactic Use of Compression Sleeves Reduces the Incidence of Arm Swelling in Women at High Risk of Breast Cancer–Related Lymphedema: A Randomized Controlled Trial*** by Paramanandam et al. 2022, please indicate your level of agreement or disagreement to the following statements.

Please see supplementary sheet for details of evidence (letters in parentheses denotes the evidence that the statement is based on).

Please indicate your level of agreement or disagreement to the following statements:

General

	Completely disagree	Disagree	Neutral	Agree	Completely agree
Prophylactic compression sleeves should be used to prevent breast cancer-related arm lymphoedema where feasible and resources allow (a)	○	○	○	○	○
Prophylactic compression sleeves is an option to prevent breast cancer-related arm lymphoedema for highly motivated patients who will comply to treatments (a)	○	○	○	○	○

Practical details of applying prophylactic compression sleeves

	Completely disagree	Disagree	Neutral	Agree	Completely agree
Prophylactic arm sleeves should be applied from first post-operative day until 3 months after the completion of adjuvant treatments (excluding hormonal treatments) (b)	○	○	○	○	○
The daily use of prophylactic arm sleeves should be at least 8 hours (c)	○	○	○	○	○
The pressure of the prophylactic arm sleeve should be between 20-25 mmHg (d)	○	○	○	○	○

Assessments when prophylactic compression sleeves are used

	Completely disagree	Disagree	Neutral	Agree	Completely agree
Patients should be assessed every 6 months for any lymphoedema while using prophylactic compression sleeves (e)	○	○	○	○	○
Patients should be assessed for breast cancer related arm lymphoedema using bioimpedance testing while using prophylactic compression sleeves (f)	○	○	○	○	○
Patients should be assessed for breast cancer related arm lymphoedema using relative volume measurements while using prophylactic compression sleeves (f)	○	○	○	○	○

Threshold for initiating subsequent treatment when prophylactic compression sleeves are used

	Completely disagree	Disagree	Neutral	Agree	Completely agree
When prophylactic compression sleeves are used, clinical lymphoedema diagnosed by bioimpedance testing or increase in relative arm volume by ≥ 10% should trigger subsequent treatments (g)	○	○	○	○	○

Part 3 - Open Responses - Prophylactic use of compression sleeves

Please state your reasons that you 'disagree' or 'strongly disagree' with the statement ***"Prophylactic compression sleeves should be used to prevent breast cancer-related arm lymphoedema where feasible and resources allow"***

Please state your reasons that you 'disagree' or 'strongly disagree' with the statement ***"Prophylactic compression sleeves is an option to prevent breast cancer-related arm lymphoedema for highly motivated patients who will comply to treatments"***

Please state your reasons that you 'disagree' or 'strongly disagree' with the statement ***"Prophylactic arm sleeves should be applied from first post-operative day until 3 months after the completion of adjuvant treatments (excluding hormonal treatments)"***

Please state your reasons that you 'disagree' or 'strongly disagree' with the statement ***"The daily use of prophylactic arm sleeves should be at least 8 hours"***

Please state your reasons that you 'disagree' or 'strongly disagree' with the statement ***"The pressure of the prophylactic arm sleeve should be between 20-25 mmHg"***

Please state your reasons that you 'disagree' or 'strongly disagree' with the statement ***"Patients should be assessed every 6 months for any lymphoedema while using prophylactic compression sleeves"***

Please state your reasons that you 'disagree' or 'strongly disagree' with the statement ***"Patients should be assessed for breast cancer related arm lymphoedema using bioimpedance testing while using prophylactic compression sleeves"***

Please state your reasons that you 'disagree' or 'strongly disagree' with the statement ***"Patients should be assessed for breast cancer related arm lymphoedema using relative volume measurements while using prophylactic compression sleeves"***

Please state your reasons that you 'disagree' or 'strongly disagree' with the statement ***"When prophylactic compression sleeves are used, clinical lymphoedema diagnosed by bioimpedance testing or increase in relative arm volume by ≥ 10% should trigger subsequent treatments"***

Part 4 - Closed Questions

Part 4 - Axillary radiation instead of axillary lymph node dissection for clinical node negative patients with positive sentinel lymph node biopsy

Based on the study ***Radiotherapy or Surgery of the Axilla After a Positive Sentinel Node in Breast Cancer: 10-Year Results of the Randomized Controlled EORTC 10981-22023 AMAROS Trial*** by Bartels et al. 2022, please indicate your level of agreement or disagreement to the following statements.

Please see supplementary sheet for details of evidence (letters in parentheses denotes the evidence that the statement is based on).

Please indicate your level of agreement or disagreement to the following statements:

	Completely disagree	Disagree	Neutral	Agree	Completely agree
Axillary radiotherapy should be given instead of axillary lymph node dissection for breast cancer patients with clinical T1 or T2, node-negative disease who are found to have positive sentinel lymph node biopsy to lower risks of lymphoedema (a)	○	○	○	○	○
Axillary radiotherapy should be offered instead of axillary lymph node dissection for breast cancer patients with clinical T1 or T2, node-negative disease who are found to have positive sentinel lymph node biopsy, regardless of the type of surgery performed (b)	○	○	○	○	○
Axillary radiotherapy should be offered instead of axillary lymph node dissection for breast cancer patients with clinical T1 or T2, node-negative disease who are found to have positive sentinel lymph node biopsy, regardless of the number of positive lymph nodes found (c)	○	○	○	○	○
Axillary radiotherapy should be offered instead of axillary lymph node dissection for breast cancer patients with clinical T1 or T2, node-negative disease who are found to have positive sentinel lymph node biopsy, regardless of the tumour grade (d)	○	○	○	○	○
Axillary radiotherapy should be offered instead of axillary lymph node dissection for breast cancer patients with clinical T1 or T2, node-negative disease who are found to have positive sentinel lymph node biopsy, regardless of hormonal receptor and HER2 status (e)	○	○	○	○	○

	Completely disagree	Disagree	Neutral	Agree	Completely agree
Axillary radiotherapy should be offered instead of axillary lymph node dissection for breast cancer patients with clinical T1 or T2, node-negative disease who are found to have positive sentinel lymph node biopsy, regardless of the number of sentinel lymph nodes removed (f)	○	○	○	○	○
When axillary radiation is recommended, the internal mammary chain should be included in the radiation portal as well (g)	○	○	○	○	○
Axillary radiotherapy should be offered instead of axillary lymph node dissection for breast cancer patients with clinical T1 or T2, node-negative disease who are found to have positive sentinel lymph node biopsy, regardless of the size of the largest sentinel lymph node (h)	○	○	○	○	○
Higher incidence of second primary cancers after axillary radiation is an important treatment consideration for young patients (age < 50) (i)	○	○	○	○	○
When breast cancer patients with clinical T1 or T2, node-negative disease receive axillary radiation after a positive sentinel lymph node biopsy, 50 Gy in 25 daily fractions is considered a suitable radiation dose fractionation (j)	○	○	○	○	○

Part 4 - Open Responses

Please state your reasons that you 'disagree' or 'strongly disagree' with the statement ***"Axillary radiotherapy should be given instead of axillary lymph node dissection for breast cancer patients with clinical T1 or T2, node-negative disease who are found to have positive sentinel lymph node biopsy to lower risks of lymphoedema"***

Please state your reasons that you 'disagree' or 'strongly disagree' with the statement ***"Axillary radiotherapy should be offered instead of axillary lymph node dissection for breast cancer patients with clinical T1 or T2, node-negative disease who are found to have positive sentinel lymph node biopsy, regardless of the type of surgery performed"***

Please state your reasons that you 'disagree' or 'strongly disagree' with the statement ***"Axillary radiotherapy should be offered instead of axillary lymph node dissection for breast cancer patients with clinical T1 or T2, node-negative disease who are found to have positive sentinel lymph node biopsy, regardless of the number of positive lymph nodes found"***

Please state your reasons that you 'disagree' or 'strongly disagree' with the statement ***"Axillary radiotherapy should be offered instead of axillary lymph node dissection for breast cancer patients with clinical T1 or T2, node-negative disease who are found to have positive sentinel lymph node biopsy, regardless of the tumour grade"***

Please state your reasons that you 'disagree' or 'strongly disagree' with the statement ***"Axillary radiotherapy should be offered instead of axillary lymph node dissection for breast cancer patients with clinical T1 or T2, node-negative disease who are found to have positive sentinel lymph node biopsy, regardless of hormonal receptor and HER2 status"***

Please state your reasons that you 'disagree' or 'strongly disagree' with the statement ***"Axillary radiotherapy should be offered instead of axillary lymph node dissection for breast cancer patients with clinical T1 or T2, node-negative disease who are found to have positive sentinel lymph node biopsy, regardless of the number of sentinel lymph nodes removed"***

Please state your reasons that you 'disagree' or 'strongly disagree' with the statement ***"When axillary radiation is recommended, the internal mammary chain should be included in the radiation portal as well"***

Please state your reasons that you 'disagree' or 'strongly disagree' with the statement ***"Axillary radiotherapy should be offered instead of axillary lymph node dissection for breast cancer patients with clinical T1 or T2, node-negative disease who are found to have positive sentinel lymph node biopsy, regardless of the size of the largest sentinel lymph node"***

Please state your reasons that you 'disagree' or 'strongly disagree' with the statement ***"Higher incidence of second primary cancers after axillary radiation is an important treatment consideration for young patients (age < 50)"***

Please state your reasons that you 'disagree' or 'strongly disagree' with the statement ***"When breast cancer patients with clinical T1 or T2, node-negative disease receive axillary radiation after a positive sentinel lymph node biopsy, 50 Gy in 25 daily fractions is considered a suitable radiation dose fractionation"***

Part 5 - Closed Questions - Prophylactic lymphatic reconstruction

Part 5 - Prophylactic lymphatic reconstruction

Based on the study ***Immediate Lymphatic Reconstruction to Prevent Breast Cancer-Related Lymphedema: A Systematic Review*** by Cook et al. 2022, please indicate your level of agreement or disagreement to the following statements.

Please see supplementary sheet for details of evidence (letters in parentheses denotes the evidence that the statement is based on).

Please indicate your level of agreement or disagreement to the following statements:

	Completely disagree	Disagree	Neutral	Agree	Completely agree
Where expertise is available and resources allow, prophylactic lymphatic reconstruction should be offered as an option to reduce risks of chronic breast cancer related lymphoedema who are unlikely able to comply to prospective surveillance programs or prophylactic use of compression sleeves (a)	○	○	○	○	○
Where expertise is available and resources allow, prophylactic lymphatic reconstruction should be offered as an option to reduce risks of chronic breast cancer related lymphoedema who have multiple concomitant high risk factors (e.g. BMI ≥ 30 kg/m2, ≥ 15 axillary lymph node dissected, plan to receive adjuvant radiotherapy) (b)	○	○	○	○	○

Part 5 - Open Responses - Prophylactic lymphatic reconstruction

Please state your reasons that you 'disagree' or 'strongly disagree' with the statement ***"Where expertise is available and resources allow, prophylactic lymphatic reconstruction should be offered as an option to reduce risks of chronic breast cancer related lymphoedema who are unlikely able to comply to prospective surveillance programs or prophylactic use of compression sleeves"***

Please state your reasons that you 'disagree' or 'strongly disagree' with the statement ***"Where expertise is available and resources allow, prophylactic lymphatic reconstruction should be offered as an option to reduce risks of chronic breast cancer related lymphoedema who have multiple concomitant high risk factors (e.g. BMI ≥ 30 kg/m2, ≥ 15 axillary lymph node dissected, plan to receive adjuvant radiotherapy)"***

Part 6 - Closed Questions - Axillary reverse mapping

Part 6 - Axillary reverse mapping

Based on the study ***Axillary Reverse Mapping in the Prevention of Lymphoedema: A Systematic Review and Pooled Analysis*** by Co et al. 2023, please indicate your level of agreement or disagreement to the following statements.

Please see supplementary sheet for details of evidence (letters in parentheses denotes the evidence that the statement is based on).

Please indicate your level of agreement or disagreement to the following statements:

	Completely disagree	Disagree	Neutral	Agree	Completely agree
Where expertise is available and resources allow, axillary reverse mapping should be offered as an option to reduce risks of chronic breast cancer related lymphoedema who are unlikely able to comply to prospective surveillance programs or prophylactic use of compression sleeves (a)	○	○	○	○	○
Axillary reverse mapping should only be offered to patients with clinical node negative disease, who are found to have a positive sentinel lymph node biopsy (b)	○	○	○	○	○
Axillary reverse mapping should only be offered to patients with clinical T1-3 disease (c)	○	○	○	○	○

Part 6 - Open Responses - Axillary reverse mapping

Please state your reasons that you 'disagree' or 'strongly disagree' with the statement ***"Where expertise is available and resources allow, axillary reverse mapping should be offered as an option to reduce risks of chronic breast cancer related lymphoedema who are unlikely able to comply to prospective surveillance programs or prophylactic use of compression sleeves"***

Please state your reasons that you 'disagree' or 'strongly disagree' with the statement ***"Axillary reverse mapping should only be offered to patients with clinical node negative disease, who are found to have a positive sentinel lymph node biopsy"***

Please state your reasons that you 'disagree' or 'strongly disagree' with the statement ***"Axillary reverse mapping should only be offered to patients with clinical T1-3 disease"***

Powered by Qualtrics
