# Supplementary material for: Multinational Association of Supportive Care in Cancer (MASCC) clinical practice guidance for the prevention of breast cancer-related arm lymphoedema (BCRAL): international Delphi consensus-based recommendations
Source: eClinicalMedicine. 2024 Feb 2;68:102441. doi: 10.1016/j.eclinm.2024.102441 (PMC10850412; doi:10.1016/j.eclinm.2024.102441)
Supplement: Supplementary information C [file mmc3.pdf]

BCRaL - Introduction / Instructions

Thank-you for consenting to participate in the study titled Multinational Association of Supportive Care in Cancer (MASCC) Led International Delphi Consensus on Measures to Prevent Breast Cancer-Related Arm Lymphoedema - Joint Project from Oncodermatology and Survivorship Study Groups.

Items that received **75% agreement** (agree and strongly agree) in Round 1 will be automatically included.

Please click here if you wish to view a summary of the changes made from Round 1 based on the overall panel feedback.

In this Round 2 survey, you will be asked to rate your agreement with the consensus statements that have been modified or newly created based on the panel's feedback in Round 1.

Once you are happy to proceed, please commence the survey by clicking on the bottom-right arrow at the bottom of the screen.

BCRaL - Participant Information

Please provide your contact details below before proceeding to the survey. These will only be used to facilitate the distribution of subsequent surveys (if required).

First Name

Surname

Email Address

Part 1 - Closed Questions - Risk for breast cancer-related arm lymphoedema

Part 1 - Risk factors for breast cancer-related arm lymphoedema

The following statements were initially based on the systematic review and meta-analysis ***Risk factors of unilateral breast cancer-related***

lymphedema: an updated systematic review and meta-analysis of 84 cohorts studies by Shen et al. 2023.

New / Modified Consensus Statements for rating in Round 2

Please indicate your level of agreement or disagreement to the following statements:

	Completely disagree	Disagree	Do not know or not within my scope of practice to judge	Agree	Completely agree
The timing of chemotherapy (neoadjuvant versus adjuvant) may impact the subsequent risks of lymphoedema but should not be a major determining factor on selecting patients for prophylactic management of lymphoedema until more studies are available.	○	○	○	○	○
The type of chemotherapy (taxane versus non-taxane) a patient receives may impact the subsequent risks of lymphoedema, but should not be a major determining factor on selecting patients for prophylactic management of lymphoedema until more studies are available.	○	○	○	○	○

Part 1 - Open Responses - Risk factors for breast cancer-related arm lymphoedema

Please state your reasons that you 'strongly disagree' or 'disagree' with the statement ***"The timing of chemotherapy (neoadjuvant versus adjuvant) may impact the subsequent risks of lymphoedema but***

should not be a major determining factor on selecting patients for prophylactic management of lymphoedema until more studies are available."

Please state your reasons that you 'strongly disagree' or 'disagree' or 'strongly disagree' with the statement ***"The type of chemotherapy (taxane versus non-taxane) a patient receives may impact the subsequent risks of lymphoedema, but should not be a major determining factor on selecting patients for prophylactic management of lymphoedema until more studies are available."***

Part 2 - Close Questions - Prospective surveillance

Part 2 - Prospective surveillance

The following statements were initially based on the study ***Prospective Surveillance for Breast Cancer–Related Arm Lymphedema: A Systematic Review and Meta-Analysis*** by Rafn et al. 2022

New / Modified Consensus Statements for rating in Round 2

Please indicate your level of agreement or disagreement to the following statements:

	Completely disagree	Disagree	Do not know or not within my scope of practice to judge	Agree	Completely agree
Bioimpedance spectroscopy is one of the more commonly used methods in the literature for early lymphoedema detection in prospective surveillance programs and can be an option before more prospective studies are available to suggest the preferred method of assessment.	○	○	○	○	○
In a prospective surveillance program, treatment is triggered when the bioimpedance spectroscopy score is L-Dex > 6.5	○	○	○	○	○
In a prospective surveillance program, treatment can be triggered when a difference in volume measurements of ≥ 5 but <10% is seen compared to pre-surgery values	○	○	○	○	○
In a prospective surveillance program, more intensive treatment is indicated (e.g., congestive decompressive therapy) when a difference in bioimpedance spectroscopy scores is L-Dex > 10 compared to pre-surgery values	○	○	○	○	○
In a prospective surveillance program, more intensive treatment (e.g., congestive decompressive therapy) is indicated when a difference in volume measurements is ≥ 10% compared to pre-surgery values	○	○	○	○	○

	Completely disagree	Disagree	Do not know or not within my scope of practice to judge	Agree	Completely agree
When subclinical / early stage lymphoedema is detected in a prospective surveillance program, compression sleeves are suggested to be prescribed for at least 4 to 6 weeks. A longer duration can be considered depending on clinical response and the individual judgement of the treating therapist.	○	○	○	○	○

Part 2 - Open Responses - Prospective surveillance

Please state your reasons that you 'strongly disagree' or 'disagree' the statement ***"Bioimpedance spectroscopy is one of the more commonly used methods in the literature for early lymphoedema detection in prospective surveillance programs and can be an option before more prospective studies are available to suggest the preferred method of assessment."***

Please state your reasons that you 'strongly disagree' or 'disagree' with the statement ***"In a prospective surveillance program, treatment is triggered when the bioimpedance spectroscopy score is L-Dex > 6.5"***

Please state your reasons that you 'strongly disagree' or 'disagree' with the statement ***"In a prospective surveillance program, treatment can be triggered when a difference in volume measurements of ≥ 5 but < 10% is seen compared to pre-surgery values"***

Please state your reasons that you 'strongly disagree' or 'disagree' with the statement ***"In a prospective surveillance program, more intensive treatment is indicated (e.g., congestive decompressive therapy) when a difference in bioimpedance spectroscopy scores is L-Dex > 10 compared to pre-surgery values"***

Please state your reasons that you 'strongly disagree' or 'disagree' with the statement ***"In a prospective surveillance program, more intensive treatment (e.g., congestive decompressive therapy) is indicated when a difference in volume measurements is ≥ 10% compared to pre-surgery values"***

Please state your reasons that you 'strongly disagree' or 'disagree' with the statement ***"When subclinical / early stage lymphoedema is detected in a prospective surveillance program, compression sleeves are suggested to be prescribed for at least 4 to 6 weeks. A longer duration can be considered depending on clinical response and the individual judgement of the treating therapist"***

Part 3 - Closed Questions - Prophylactic use of compression sleeves

Part 3 - Prophylactic use of compression sleeves

The following statements were initially based on the study ***Prophylactic Use of Compression Sleeves Reduces the Incidence of Arm Swelling in Women at High Risk of Breast Cancer–Related Lymphedema: A Randomized Controlled Trial*** by Paramanandam et al. 2022.

New / Modified Consensus Statements for rating in Round 2

Please indicate your level of agreement or disagreement to the following statements:

	Strongly disagree	Disagree	Do not know or not within my scope of practice to judge	Agree	Completely agree
Prophylactic compression sleeves should be offered as an option to prevent breast cancer-related arm lymphoedema.	○	○	○	○	○
For patients at high risk of lymphedema who wish to consider prophylactic arm sleeves, the sleeves should be applied from the first post-operative day until 3 months after the completion of adjuvant treatments (excluding hormonal treatments).	○	○	○	○	○
The daily use of prophylactic arm sleeves is suggested to be at least 8 hours. The duration should be individualized depending on patients' preferences and comfort while wearing the sleeves.	○	○	○	○	○
The pressure of the prophylactic arm sleeve should be reviewed regularly and adjusted to patients' risks and needs.	○	○	○	○	○

Part 3 - Open Responses - Prophylactic use of compression sleeves

Please state your reasons that you 'strongly disagree' or 'disagree' with the statement ***"Prophylactic compression sleeves should be offered as an option to prevent breast cancer-related arm lymphoedema"***

[ ]

Please state your reasons that you 'strongly disagree' or 'disagree' with the statement ***"For patients at high risk of lymphedema who wish to consider prophylactic arm sleeves, the sleeves should be applied from the first post-operative day until 3 months after the completion of adjuvant treatments (excluding hormonal treatments)"***

Please state your reasons that you 'strongly disagree' or 'disagree' with the statement ***"The daily use of prophylactic arm sleeves is suggested to be at least 8 hours. The duration should be individualized depending on patients' preferences and comfort while wearing the sleeves"***

Please state your reasons that you 'strongly disagree' or 'disagree' with the statement ***"The pressure of the prophylactic arm sleeve should be reviewed regularly and adjusted to patients' risks and needs"***

Part 4 - Closed Questions

Part 4 - Axillary radiation instead of axillary lymph node dissection for clinical node negative patients with positive sentinel lymph node biopsy

The following statements were initially based on the study ***Radiotherapy or Surgery of the Axilla After a Positive Sentinel Node in Breast Cancer: 10-Year Results of the Randomized Controlled EORTC 10981-22023 AMAROS Trial*** by Bartels et al. 2022.

New / Modified Consensus Statements for rating in Round 2

Please indicate your level of agreement or disagreement to the following statements:

	Completely disagree	Disagree	Do not know or not within my scope of practice to judge	Agree	Completely agree
Axillary lymph node dissection should not be routinely offered to breast cancer patients with clinical T1 or T2, node-negative disease who are found to have 1 to 2 positive sentinel lymph nodes and received breast conservation therapy.	○	○	○	○	○
Axillary radiotherapy instead of axillary lymph node dissection can be considered in breast cancer patients with clinical T1 or T2, node-negative disease who are found to have 1 to 2 positive sentinel lymph nodes and received mastectomy.	○	○	○	○	○
Axillary lymph node dissection should be offered instead of axillary radiation in breast cancer patients with clinical T1 or T2, node-negative disease who are found to have more than 2 positive sentinel lymph nodes.	○	○	○	○	○

	Completely disagree	Disagree	Do not know <u>or</u> not within my scope of practice to judge	Agree	Completely agree
For clinical T1 or T2, node-negative breast cancer patients with high risk tumour biology (e.g., triple negative, grade 3) who are found to have 1 to 2 positive sentinel lymph nodes, an individualized decision should be made with the patient whether to perform axillary dissection or give axillary radiation.	○	○	○	○	○
In clinical T1 or T2, node-negative disease who are found to have positive sentinel lymph nodes with less than 2 lymph nodes removed or having extra-nodal extension, an individualized decision should be made with the patient whether to perform axillary dissection or give axillary radiation.	○	○	○	○	○
When axillary radiation is recommended in clinical T1-2 node negative breast cancer with a positive sentinel lymph node, the decision to include internal mammary chain in the radiation portal should be individualized depending on factors such as location of primary tumour and presence of cardiac risk factors.	○	○	○	○	○
A relatively higher incidence of second primary cancers after axillary radiation should be highlighted in young patients (age < 50), but should not affect the decision to offer radiotherapy if indicated.	○	○	○	○	○
In accordance with international guidelines, moderate hypofractionation (40 to 42.5 Gy in 15-16 fractions) is preferred over 50 Gy in 25 fractions when axillary radiotherapy is given to clinical T1 or T2, node-negative patients with a positive sentinel lymph node biopsy.	○	○	○	○	○

Part 4 - Open Responses

Please state your reasons that you 'strongly disagree' or 'disagree' with the statement ***"Axillary lymph node dissection should not be routinely offered to breast cancer patients with clinical T1 or T2, node-negative disease who are found to have 1 to 2 positive sentinel lymph nodes and received breast conservation therapy"***

Please state your reasons that you 'strongly disagree' or 'disagree' with the statement ***"Axillary radiotherapy instead of axillary lymph node dissection can be considered in breast cancer patients with clinical T1 or T2, node-negative disease who are found to have 1 to 2 positive sentinel lymph nodes and received mastectomy"***

Please state your reasons that you 'strongly disagree' or 'disagree' with the statement ***"Axillary lymph node dissection should be offered instead of axillary radiation in breast cancer patients with clinical T1 or T2, node-negative disease who are found to have more than 2 positive sentinel lymph nodes"***

Please state your reasons that you 'strongly disagree' or 'disagree' with the statement ***"For clinical T1 or T2, node-negative breast cancer patients with high risk tumour biology (e.g., triple negative, grade 3) who are found to have 1 to 2 positive sentinel lymph nodes, an individualized decision should be made with the patient whether to perform axillary dissection or give axillary radiation"***

Please state your reasons that you 'strongly disagree' or 'disagree' with the statement ***"In clinical T1 or T2, node-negative disease who are found to have positive sentinel lymph nodes with less than 2 lymph nodes removed or having extra-nodal extension, an individualized decision should be made with the patient whether to perform axillary dissection or give axillary radiation"***

Please state your reasons that you 'strongly disagree' or 'disagree' with the statement ***"When axillary radiation is recommended in clinical T1-2 node negative breast cancer with a positive sentinel lymph node, the decision to include internal mammary chain in the radiation portal should be individualized depending on factors such as location of primary tumour and presence of cardiac risk factors"***

Please state your reasons that you 'strongly disagree' or 'disagree' with the statement ***"A relatively higher incidence of second primary cancers after axillary radiation should be highlighted in young patients (age < 50), but should not affect the decision to offer radiotherapy if indicated"***

Please state your reasons that you 'strongly disagree' or 'disagree' with the statement ***"In accordance with international guidelines, moderate hypofractionation (40 to 42.5 Gy in 15-16 fractions) is preferred over 50 Gy in 25 fractions when axillary radiotherapy is given to clinical T1 or T2, node-negative patients with a positive sentinel lymph node biopsy"***

Part 5 - Closed Questions - Prophylactic lymphatic reconstruction

Part 5 - Prophylactic lymphatic reconstruction

The following questions were initially based on the study ***Immediate***

Lymphatic Reconstruction to Prevent Breast Cancer-Related Lymphedema: A Systematic Review by Cook et al. 2022.

New / Modified Consensus Statements for rating in Round 2

Please indicate your level of agreement or disagreement to the following statements:

	Completely disagree	Disagree	Do not know or not within my scope of practice to judge	Agree	Completely agree
Where expertise is available and resources allow, prophylactic lymphatic reconstruction is an option to reduce risks of chronic breast cancer related lymphoedema in patients who require extensive axillary lymph node dissection for large or multiple clinically positive lymph nodes.	○	○	○	○	○

Part 5 - Open Responses - Prophylactic lymphatic reconstruction

Please state your reasons that you 'strongly disagree' or 'disagree' with the statement ***"Where expertise is available and resources allow, prophylactic lymphatic reconstruction is an option to reduce risks of chronic breast cancer related lymphoedema in patients who require extensive axillary lymph node dissection for large or multiple clinically positive lymph nodes"***

Part 6 - Closed Questions - Axillary reverse mapping

Part 6 - Axillary reverse mapping

The following questions were initially based on the study ***Axillary Reverse Mapping in the Prevention of Lymphoedema: A Systematic Review and Pooled Analysis*** by Co et al. 2023.

New / Modified Consensus Statements for rating in Round 2

Please indicate your level of agreement or disagreement to the following statements:

	Completely disagree	Disagree	Do not know or not within my scope of practice to judge	Agree	Completely agree
Where expertise is available and resources allow, axillary reverse mapping is an option for patients indicated for axillary lymph node dissection to reduce risks of chronic breast cancer related lymphoedema.	○	○	○	○	○
Axillary reverse mapping should not be offered in patients at high risk of axillary recurrence (e.g., multiple clinically positive lymph nodes, T4 primary).	○	○	○	○	○

Part 6 - Open Responses - Axillary reverse mapping

Please state your reasons that you 'strongly disagree' or 'disagree' with the statement ***"Where expertise is available and resources allow, axillary reverse mapping is an option for patients indicated for axillary lymph node dissection to reduce risks of chronic breast cancer related lymphoedema"***

Please state your reasons that you 'strongly disagree' or 'disagree' with the statement ***"Axillary reverse mapping should not be offered in patients at high risk of axillary recurrence (e.g., multiple clinically positive lymph nodes, T4 primary)"***

Powered by Qualtrics
