# Supplementary material for: Multinational Association of Supportive Care in Cancer (MASCC) clinical practice guidance for the prevention of breast cancer-related arm lymphoedema (BCRAL): international Delphi consensus-based recommendations
Source: eClinicalMedicine. 2024 Feb 2;68:102441. doi: 10.1016/j.eclinm.2024.102441 (PMC10850412; doi:10.1016/j.eclinm.2024.102441)
Supplement: Appendix 1 [file mmc4.docx]

**Appendix 1: Multinational Association In Supportive Care in Cancer (MASCC) Breast Cancer Related Arm Lymphoedema (BCRAL) Expert Panel**

| **Name** | **Position** | **Affiliation** | **Location of Practice** | **Primary occupation** |
| --- | --- | --- | --- | --- |
| Muna AlKhaifi | Assistant Professor | Department of Medical Oncology, Sunnybrook Health Sciences Centre | Toronto, Canada | General Practitioner Oncologist (GPO) |
| Belen Alonso Alvarez | Rehabilitation Physician | Department of Physical Medicine and Rehabilitation, Hospital Universitario Ramón y Cajal | Madrid, Spain | Rehabilitation Physician |
| Suvam Banerjee | Medical doctor and researcher | Burdwan Medical College and Hospital | Bardhhaman, India | General Practitioner |
| Kira Bloomquist | Postdoctoral Research Fellow | University Hospitals Center for Health Research, Copenhagen University Hospital, Rigshospitalet | Copenhagen, Denmark | Physiotherapist |
| Pierluigi Bonomo | Radiation Oncologist | Azienda Ospedaliero-Universitaria Careggi, University of Florence | Florence, Italy | Radiation Oncologist |
| Pinar Borman | Professor | Department of Physical Medicine and Rehabilitation, University of Health Sciences Ankara City Hospital | Ankara, Turkey | Rehabilitation physician |
| Yolande Borthwick | Lecturer | School of Medicine, Dentistry & Nursing, University of Glasgow | Glasgow, U.K. | Lymphoedema Specialist |
| Dominic Chan | Consultant | Department of Oncology, Princess Margaret Hospital | Hong Kong S.A.R, China | Clinical Oncologist |
| Sze Man Chan | Associate Nursing Consultant | Department of Clinical Oncology, Pamela Youde Nethersole Eastern Hospital | Hong Kong S.A.R, China | Nurse |
| Yolanda Chan | Consultant | CUHK Medical Centre | Hong Kong S.A.R, China | Surgeon |
| Ngan Sum Jean Cheng | Associate Consultant | Department of Clinical Oncology, Tuen Mun Hospital | Hong Kong S.A.R, China | Clinical Oncologist |
| J. Isabelle Choi | Assistant Attending Radiation Oncologist | Department of Radiation Oncology, Memorial Sloan Kettering Cancer Center | New York City, New York, USA | Radiation Oncologist |
| Edward Chow | Professor | Department of Radiation Oncology, University of Toronto | Toronto, Canada | Radiation Oncologist |
| Yin Ping Choy | Nursing Consultant | Department of Oncology, Princess Margaret Hospital | Hong Kong S.A.R, China | Nurse |
| Kimberly Corbin | Associate Professor of Radiation Oncology | Department of Radiation Oncology, Mayo Clinic | Rochester, Minnesota, USA | Radiation Oncologist |
| Elizabeth Dylke | Associate Professor | Faculty of Medicine and Health, University of Sydney | Sydney, Australia | Researcher |
| Pamela Hammond | Registered Massage Therapist, Certified Combined Decongestive Therapist | Princess Margaret Cancer Centre, The University of Toronto | Toronto, Canada | Massage therapist |
| Satoshi Hirakawa | Dermatologist | Department of Supportive Care in Cancer, Seirei Hamamatsu General Hospital | Hamamatsu, Japan | Dermatologist |
| Kimiko Hirata | Radiation Oncologist | Department of Radiation Oncology, Kyoto City Hospital | Kyoto, Japan | Radiation Oncologist |
| Shing Fung Lee | Associate Consultant | Department of Radiation Oncology, National University Cancer Institute, National University Hospital, Singapore | Singapore | Radiation Oncologist |
| Marianne Holt | Physiotherapist | Department of Oncology, Odense University Hospital | Odense, Denmark | Physiotherapist |
| Peter Johnstone | Professor | Department of Radiation Oncology, Mofitt Cancer Center | Tampa, Florida, USA | Radiation Oncologist |
| Yuichiro Kikawa | Breast Surgeon | Department of Breast Surgery, Kansai Medical University Hospital | Osaka, Japan | Surgeon |
| Deborah Kirk | Associate Professor | School of Nursing and  Midwifery, Edith Cowan  University | Perth, Australia | Researcher |
| Haruru Kotani | Chief Physician | Aichi Cancer Center Hospital | Nagoya, Japan | Surgeon |
| Carol Kwok | Consultant | Department of Oncology, Princess Margaret Hospital, Hong Kong | Hong Kong S.A.R, China | Clinical Oncologist |
| Jessica Lai | Consultant | Department of Oncology, Princess Margaret Hospital | Hong Kong S.A.R, China | Clinical Oncologist |
| Mei Ying Lim | Consultant | Department of Oncology, Princess Margaret Hospital | Hong Kong S.A.R, China | Clinical Oncologist |
| Michael Lock | Professor | Department of Radiation Oncology, University of Western Ontario, Canada | London, Canada | Radiation Oncologist |
| Brittany Lorden | Occupational Therapist Oncology Clinical Specialist, Certified Lymphedema Therapist | Department of Supportive Oncology, Atrium Health Levine Cancer Institute | Charlotte, North Carolina, USA | Occupational Therapist |
| Page Mack | Physical Therapist, Certified Lymphedema Therapist | Department of Supportive Oncology, Atrium Health Levine Cancer Institute | Charlotte, North Carolina, USA | Physical Therapist |
| Stefano Magno | Breast Surgeon | Fondazione Policlinico Universitario Gemelli IRCCS | Rome, Italy | Surgeon |
| Icro Meattini | Associate Professor | Department of Radiation Oncology, Florence University | Florence, Italy | Radiation Oncologist |
| Gustavo Nader Marta | Professor | 1. Department of Radiation Oncology, Hospital Sírio- Libanês, Sao Paulo, Brazil.  2. Latin America Cooperative Oncology Group, Porto Alegre, Brazil  3. Post-Graduation Program. Department of Radiology and Oncology - Faculdade de Medicina FMUSP, Universidade de Sao Paulo, Sao Paulo, Brazil; | Sao Paulo, Brazil | Radiation Oncologist |
| Margaret McNeely | Professor | Faculty of Rehabilitation Medicine, University of Alberta | Edmonton, Canada | Researcher |
| Tammy Mondry | Physical Therapist | Cancer Therapy Specialist | San Diego, California, USA | Physiotherapist |
| Luis Enrique Lopez Montoya | Professor | Fi Fisioterapia Integral S.C. Research Group | Zapopan, Mexico | Physiotherapist |
| Mami Ogita | Assistant Professor | Department of Radiology, University of Tokyo Hospital | Tokyo, Japan | Radiation Oncologist |
| Misato Osaka | Nursing Specialist | Medical Oncology Department, St. Luke's International Hospital | Tokyo, Japan | Nurse |
| Stephanie Phan | Clinical Lead | Cancer Rehabilitation and Survivorship Program, Department of Supportive Care, Princess Margaret Cancer Centre, The University of Toronto, Toronto | Toronto, Canada | Occupational Therapist |
| Philip Poortmans | Associate Professor | Department of Radiation Oncology, Iridium Network and Faculty of Medicine and Health Sciences, University of Antwerp | Antwerp, Belgium | Radiation Oncologist |
| Bolette Skjødt Rafn | Physical therapist, Post-doctorate Researcher | Department of Oncology, Copenhagen University Hospital | Copenhagen, Denmark | Researcher |
| Abram Recht | Professor | Department of Radiation Oncology, Harvard Medical School | Boston, Massachusetts, USA | Radiation Oncologist |
| Agata Rembielak | Professor | 1. The Christie NHS Foundation Trust  2. The University of Manchester | Manchester, U.K. | Clinical Oncologist |
| Angela Río-González | Professor | 1. Spanish Lymphoedema Association  2. Universidad Europea de Madrid | Madrid, Spain | Physiotherapist |
| Jolien Robijns | Postdoctoral Researcher | 1. Faculty of Medicine and Life Sciences, Hasselt University, Limburg Clinical Research Centre  2. Department of Oncology & Radiation Therapy and Department of Jessa & Science, Jessa Hospital | Hasselt, Belgium | Researcher |
| Naoko Sanuki | Radiation Oncologist | Department of Radiology, Yokkaichi Municipal Hospital | Yokkaichi, Japan | Radiation Oncologist |
| Charles B. Simone, II | Chief Medical Officer | New York Proton Center | New York City, New York, USA | Radiation Oncologist |
| Mateusz Spałek | Deputy Head | Department of Radiotherapy, Maria Sklodowska-Curie National Research Institute of Oncology | Warsaw, Poland | Radiation Oncologist |
| Kaori Tane | Chief Physician | Department of Breast Surgery, Hyogo Cancer Center | Hyogo, Japan | Surgeon |
| Luiz Felipe Nevola Teixeira | Physiotherapist | Istituto Europeo di Oncologia | Milan, Italy | Physiotherapist |
| Mitsuo Terada | Clinical Assistant Professor | Department of Breast Surgery, Graduate School of Medical Sciences, Nagoya City University | Nagoya, Japan | Surgeon |
| Mark Trombetta | Professor | Division of Radiation Oncology, Drexel University College of Medicine | Philadelphia, Pennsylvania, USA | Radiation Oncologist |
| Kam Hung Wong | Consultant | Department of Clinical Oncology, Queen Elizabeth Hospital | Hong Kong S.A.R, China | Clinical Oncologist |
| Katsuhide Yoshidome | Director | Breast and Endocrine Surgery, Osaka Police Hospital | Osaka, Japan | Surgeon |
