# Supplementary material for: Multinational Association of Supportive Care in Cancer (MASCC) clinical practice guidance for the prevention of breast cancer-related arm lymphoedema (BCRAL): international Delphi consensus-based recommendations
Source: eClinicalMedicine. 2024 Feb 2;68:102441. doi: 10.1016/j.eclinm.2024.102441 (PMC10850412; doi:10.1016/j.eclinm.2024.102441)
Supplement: Expert Panel Names [file mmc5.docx]

**Multinational Association in Supportive Care in Cancer (MASCC) Breast Cancer Related Arm Lymphoedema (BCRAL) Expert Panel**

| **First names** | **Surnames** |
| --- | --- |
| Muna | AlKhaifi |
| Belen Alonso | Alvarez |
| Suvam | Banerjee |
| Kira | Bloomquist |
| Pierluigi | Bonomo |
| Pinar | Borman |
| Yolande | Borthwick |
| Dominic | Chan |
| Sze Man | Chan |
| Yolanda | Chan |
| Ngan Sum Jean | Cheng |
| J. Isabelle | Choi |
| Edward | Chow |
| Yin Ping | Choy |
| Kimberly | Corbin |
| Elizabeth | Dylke |
| Pamela | Hammond |
| Satoshi | Hirakawa |
| Kimiko | Hirata |
| Shing Fung | Lee |
| Marianne | Holt |
| Peter | Johnstone |
| Yuichiro | Kikawa |
| Deborah | Kirk |
| Haruru | Kotani |
| Carol | Kwok |
| Jessica | Lai |
| Mei Ying | Lim |
| Michael | Lock |
| Brittany | Lorden |
| Page | Mack |
| Stefano | Magno |
| Icro | Meattini |
| Gustavo Nader | Marta |
| Margaret | McNeely |
| Tammy | Mondry |
| Luis Enrique Lopez | Montoya |
| Mami | Ogita |
| Misato | Osaka |
| Stephanie | Phan |
| Philip | Poortmans |
| Bolette Skjødt | Rafn |
| Abram | Recht |
| Agata | Rembielak |
| Angela | Río-González |
| Jolien | Robijns |
| Naoko | Sanuki |
| Charles B. | Simone, II |
| Mateusz | Spałek |
| Kaori | Tane |
| Luiz Felipe Nevola | Teixeira |
| Mitsuo | Terada |
| Mark | Trombetta |
| Kam Hung | Wong |
| Katsuhide | Yoshidome |
